# The Design of FluxML: A Universal Modeling Language for ^13^C Metabolic Flux Analysis

**DOI:** 10.3389/fmicb.2019.01022

**Published:** 2019-05-24

**Authors:** Martin Beyß, Salah Azzouzi, Michael Weitzel, Wolfgang Wiechert, Katharina Nöh

**Affiliations:** ^1^Institute of Bio- and Geosciences, IBG-1: Biotechnology, Forschungszentrum Jülich GmbH, Jülich, Germany; ^2^Computational Systems Biotechnology (AVT.CSB), RWTH Aachen University, Aachen, Germany

**Keywords:** ^13^C metabolic flux analysis, FluxML, machine-readable format, model specification language, computational modeling, reproducible science, data models, model exchange

## Abstract

^13^C metabolic flux analysis (MFA) is the method of choice when a detailed inference of intracellular metabolic fluxes in living organisms under metabolic quasi-steady state conditions is desired. Being continuously developed since two decades, the technology made major contributions to the quantitative characterization of organisms in all fields of biotechnology and health-related research. ^13^C MFA, however, stands out from other “-omics sciences,” in that it requires not only experimental-analytical data, but also mathematical models and a computational toolset to infer the quantities of interest, i.e., the metabolic fluxes. At present, these models cannot be conveniently exchanged between different labs. Here, we present the implementation-independent model description language FluxML for specifying ^13^C MFA models. The core of FluxML captures the metabolic reaction network together with atom mappings, constraints on the model parameters, and the wealth of data configurations. In particular, we describe the governing design processes that shaped the FluxML language. We demonstrate the utility of FluxML to represent many contemporary experimental-analytical requirements in the field of ^13^C MFA. The major aim of FluxML is to offer a sound, open, and future-proof language to unambiguously express and conserve all the necessary information for model re-use, exchange, and comparison. Along with FluxML, several powerful computational tools are supplied for easy handling, but also to maintain a maximum of flexibility. Altogether, the FluxML collection is an “all-around carefree package” for ^13^C MFA modelers. We believe that FluxML improves scientific productivity as well as transparency and therewith contributes to the efficiency and reproducibility of computational modeling efforts in the field of ^13^C MFA.

## Introduction

Systems Biology combines high-throughput experimentation with quantitative analysis and computational modeling to approach an understanding on how cellular phenotypes emerge from molecular interactions (Wolkenhauer, 2001; Westerhoff and Hofmeyr, 2005). To this end, a comprehensive set of “omics” techniques has been developed ranging from transcriptomics, proteomics, metabolomics to fluxomics, the quantification of metabolic reaction rates (fluxes) *in vivo* (Nielsen, 2003). In the field of fluxomics, metabolic flux analysis (MFA) with stable isotope tracers, typically a ^13^C labeled carbon source, is being considered as the “gold standard” for flux quantification under metabolic quasi-steady state conditions (Wiechert, 2001; Sauer, 2006). Being systematically developed in the mid-1990s (Marx et al., 1996; Christensen and Nielsen, 1999), ^13^C MFA has been applied to a wide variety of organisms (microbes, plants, mammalian cell lines), cultivated under different conditions (chemostat, batch, fed-batch), in single, co-culture and host-pathogen systems (Beste et al., 2013; Ghosh et al., 2014; Gebreselassie and Antoniewicz, 2015), and probed with diverse labeling strategies (e.g., ^13/14^C, ^2^H, ^15^N)^1^ within isotopic transient or steady-state regimes (Zamboni et al., 2009; Niedenführ et al., 2015; Allen, 2016; Schwechheimer et al., 2018). For introductory texts on ^13^C MFA, the reader is referred to the literature (Zamboni et al., 2009; Wiechert et al., 2015; Dai and Locasale, 2017).

Direct procedures to measure fluxes exist solely for extracellular rates, i.e., uptake and secretion fluxes. The determination of intracellular fluxes *in vivo* requires two additional ingredients: First, the measurement of the labeling incorporation into the intracellular metabolites. To this end, various analytical techniques such as homo- or heteronuclear, scalar- or multi-dimensional nuclear magnetic resonance (NMR) as well as single or tandem mass spectrometry (MS) are nowadays applied (Wittmann and Heinzle, 2001; Luo et al., 2007; Lane et al., 2008; Yuan et al., 2010; Giraudeau et al., 2011; Blank et al., 2012; Chu et al., 2015; Kappelmann et al., 2017). Second, and in contrast to other omics technologies, a powerful computational machinery is mandatory for data evaluation and flux inference. This means that the measured information, i.e., the isotopic data of intracellular metabolites together with the extracellular rates, does not directly uncover the desired flux information. The relation between isotopic enrichments and the fluxes is captured in a mathematical model which predicts the emerging fractional labeling patterns from given flux values. Clearly, this model has to be operated in the inverse direction to infer the, in reality, unknown fluxes from the observed data. These fluxes are then determined in an iterative fitting procedure in which the log-likelihood function, expressing the discrepancies between the model-predicted and measured quantities, is minimized. Finally, statistical measures estimate the confidence with which the fluxes are inferred from the data in view of their precision (Wiechert et al., 1997; Theorell et al., 2017).

As a consequence of this procedure, the results of any ^13^C MFA intimately depend on the metabolic network model used. Metabolic networks for ^13^C MFA heavily vary in size, from focused representations consisting of only a few tens of reaction steps (Zamboni et al., 2009) to comprehensive descriptions with hundreds of reactions (Gopalakrishnan and Maranas, 2015; McCloskey et al., 2016b). Since the flux estimation procedure with such networks is computationally demanding, a number of algorithms have been proposed over the last two decades to speed up the core computation steps (Wiechert et al., 1999; Zamboni et al., 2005; Antoniewicz et al., 2007; Weitzel et al., 2007; Tepper and Shlomi, 2015). Unsurprisingly, these developments have led to the emergence of a variety of software tools that are almost as diverse as the experimental scenarios of ^13^C MFA (see Supplementary S1 Table 1.1).

More on the ^13^C MFA methodology and the assortment of flux analysis methods being applied is found elsewhere in the literature (Zamboni et al., 2009; Niedenführ et al., 2015; Wiechert et al., 2015). For the following considerations, it is sufficient to recognize that ^13^C MFA in practice means a combinatorial variety of possible experimental, analytical, and computational configurations as well as model incarnations. The pros and cons of these different frameworks should not be scrutinized here. However, one aspect has to be emphasized: Despite of the heterogeneity of use cases, there is little debate about the principal conditions under which a ^13^C MFA experiment must be conducted (i.e., metabolic pseudo-stationarity, homogeneous cell populations), and the input required for setting up the computational model (e.g., the structural description of the biochemical network underlying the model, specification of tracers, and measurements). Consequently, the precise configuration for an individual case comprises lots of specific details about the experimental-analytical setup and is, as evidenced in Section FluxML IN A NUTSHELL, rather complex.

### Why Is There a Need for a Standardized Model Exchange Format in ^13^C MFA?

Bundling all the aspects specific to an individual ^13^C MFA study in a standardized document is undoubtedly of tremendous value for the community. This has already been proven by the success of the Systems Biology Markup Language (SBML, Hucka et al., 2003), which is today used as *lingua franca* to handle model-exchange between hundreds of different computational systems biology tools, as well as various other established modeling languages such as CellML (Lloyd et al., 2004) and NeuroML (Gleeson et al., 2010). Transferred to the ^13^C MFA domain, this means to have a flux document formulated in a universal, i.e., network, algorithm-, tool-, and measurement-independent modeling language that is governed by controlled vocabularies and covers all current application cases.

A universal ^13^C MFA modeling language allows sharing and publishing models in a complete, unambiguous, and re-usable way. At present, this is only wishful thinking as existing guidelines (Crown and Antoniewicz, 2013) are, as we argue, not sufficiently strict. As a result, published papers do almost never supply all the information required to enable full reproduction of the model(s) used in the study. Partly, this incompleteness is due to the configuration processes that are too complex for full reproduction in a paper. But also, implicit assumptions made in the modeling process—either by the modeler or hidden in the encoding of the software tool—remain undocumented, maybe unintentionally. In this sense, a standardized ^13^C MFA modeling language provides a rule set to scientists for reporting re-usable models.

**Figure 1:**
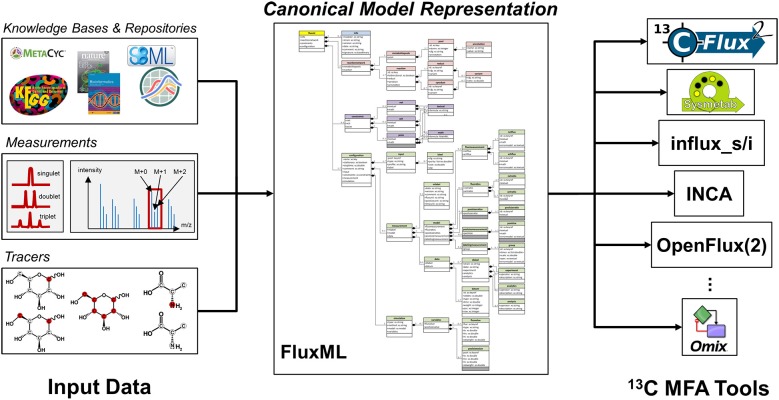
Central role of a canonical model representation for ^13^C MFA.

In a wider context, model exchange formats are an essential component for the reproduction of simulation results within the complex computational pipelines (Ebert et al., 2012; Dalman et al., 2016). As a practical benefit, a ^13^C MFA modeling language empowers modelers to concentrate on the specification of the underlying network model, independent of the specific implementation in a software tool (cf. Figure 1). Such an “Esperanto” format is, thus, the central component for serving the FAIR Data Principles (Wilkinson et al., 2016). To put it straight, a standardized model exchange format fills the void and resolves many, if not all, of the current deficiencies. In addition, it paves the way for enhancing the models' shelf lifes and increases the efficiency of modeling efforts.

In this work we discuss the question: How should a universal model specification look like that digitally codifies all data required to carry out a ^13^C MFA? By expanding on our former work (Wiechert et al., 2001), we motivate the benefits of a modern computer readable markup language for ^13^C MFA, called Flux Markup Language (FluxML), and describe the governing principles of its design. To this end, we work out the required content that constitutes a model, formally known as *syntax standard*. Here, special focus is given to the model-data integration and extensibility aspects to keep pace with ongoing experimental-analytical developments. Clearly, to be adopted, such a general model representation effort must be accompanied by a set of supporting tools facilitating validation and modification tasks. We supply several computational tools with the modeling language, making the FluxML collection an “all-around carefree package” for modelers. The collection is illustrated with typical ^13^C MFA examples at hand: First, we demonstrate that the FluxML model format unlocks the comparability of state-of-the-art simulators, an aspect that is dearly missing, even 20 years after advent of the first simulators. Secondly, we illustrate how easy the configuration task of parallel labeling experiments is with FluxML.

## A Content Standard for the Exchange of Models and Data in ^13^C MFA

A model exchange document has to encapsulate all (necessary and optional) components, their interconnections, and the parameter information from which the computational model is built. In addition, since ^13^C MFA is an experimental method, experimental data descriptors have to be defined from which the fluxes are to be inferred. To illustrate this in more detail, the specification of an isotope labeling experiment (ILE) and the corresponding measurement data includes the following elements (Wiechert, 2001; Wiechert et al., 2001):

1. Reaction stoichiometry, i.e., the structure of the metabolic model that defines the scope of the flux analysis. Depending on the desired resolution, metabolic pathways are formulated in full detail or as simplified, lumped reaction chains.
2. For modeling the flow of the relevant isotope tracer through the metabolic pathways, atom transitions for each reaction step, e.g., carbon atom transitions in case of ^13^C labeling or carbon and nitrogen transitions in case of simultaneous ^13^C-^15^N tracers. The atom transitions specify the precise mapping between the atoms of the reactions' substrates and products. For reactants with rotation-symmetrical molecular structure multiple combinatorial atom mapping possibilities ensue (*scrambling reactions*).
3. Settings for metabolic fluxes as, for example, reaction directionality, range restrictions to keep flux values in physiologically sensible or desired limits, or to constrain believed-equal fluxes through scrambling reactions.
4. Tracer composition of the substrate or substrate mixture used for the ILE. This includes the exchange of intracellular metabolites with isotopically unlabeled external ones (such as rich media components, CO_2_ etc.).
5. The types of measurements that are attained from the ILE (or, at the stage of a priori experimental design, are envisioned to be attainable), i.e., the *measurement configuration*:
  - Extracellular rates (or external fluxes), as derived from concentration profiles of exometabolites or bioprocess models.
  - Fractional labeling enrichments obtained by analytical instruments (e.g., positional labeling, mass isotopomer fragments, multiplets, etc.). For isotopically steady state conditions one set of labeling data is specified, while under isotopically non-stationary conditions (INST ^13^C MFA) a time series of such sets is to be integrated representing the transient incorporation of label.
  - Intracellular pool sizes (i.e., concentrations) are key determinants of the labeling incorporation velocity and should, thus, be specified in INST ^13^C MFA if experimentally accessible (Wiechert and Nöh, 2005; Nöh et al., 2006).
  Importantly, each measurement must be accompanied with an associated standard deviation quantifying its precision.
6. A set of variables that parametrizes the underlying computational model and enables its execution, e.g., the set of free fluxes (Wiechert and de Graaf, 1996).

This list can be regarded as minimal content standard for ^13^C MFA models in the notion of Minimum Information Requested In the Annotation of biochemical Models (MIRIAM) (Le Novère et al., 2005). However, to define a language, a *syntax standard* (or *format*) is needed that provides structures for formatting the information laid down in the content standard. In addition, terminology and rules to specify valid models have to be declared, therewith enabling the semantic interpretation of the model descriptions.

The requirement to deal with the broad diversity of ^13^C MFA options renders the design of a modeling language a challenging endeavor. Before discussing the design decisions for FluxML in detail, former developments in the computational field should be briefly reviewed.

### A Short History of ^13^C MFA Modeling

Software systems developed in the past have used different approaches to supply the information needed to execute ^13^C MFA. Several of the first generation flux analysis tools developed in the 90ies did not rely on dedicated specification formats but rather formulate the network and associated measurements by a set of matrices: atom mapping matrices to describe atom transitions (Zupke and Stephanopoulos, 1994) or isotopomer mapping matrices that unfold the system of isotopomer balance equations (Schmidt et al., 1997). One obvious problem with this matrix-centered approach is that it is prone to introduce specification errors which are hardly detectable afterwards.

To overcome this weakness, many second generation tools such as FiatFlux (Zamboni et al., 2005), tcaSIM/tcaCALC (Sherry et al., 2004), Metran (Young et al., 2008), INCA (Young, 2014), or WuFlux (He et al., 2016) have been equipped with graphical user interfaces (GUI) for a convenient model formulation (cf. Supplementary S1 Table 1.1). Such solutions are designed with having the end-user, typically an experimentalist, in mind who does not want to care about too many technical details. While the user-friendliness of these GUI-based tools is unraveled, they come at the price of a substantially restricted modeling flexibility: the abilities to change the reaction network or to formulate different measurement configurations are rather limited.

The first software framework for ^13^C MFA that was able to deal with any isotopically stationary experimental setup in a freely configurable manner was 13CFLUX (Wiechert et al., 2001). Owing to the popularity of spreadsheets among experimentalists, 13CFLUX relies on tabulator-delimited text files for model and data specification, the FTBL (*Flux TaBuLar*) format. FTBLs' concept to divide the required information into several contextual sections has been adopted by many software packages such as OPENFLUX(2) (Quek et al., 2009; Shupletsov et al., 2014), FIA (Srour et al., 2011), and influx_s(i) (Sokol et al., 2012).

Despite the widespread use of FTBL, recent trends for automated lab experimentation and computational analysis pipelines (Dalman et al., 2010, 2016; Heux et al., 2017) call for contemporary model specification formats that are computationally easier to access and better verifiable than spreadsheets. Consequently, with our second generation ^13^C MFA software 13CFLUX2 (Weitzel et al., 2013) an update to FTBL was proposed: the Flux Markup Language FluxML. FluxML exploits the powerful eXtensible Markup Language (XML) framework which has been designed to ease the computational processing of structured text documents. However, at the time of its publication, FluxML supported exclusively the formulation of isotopic stationary ^13^C MFA models.

## Decisions on the Design of FluxML

### Universal ^13^C MFA Model Exchange Formats—Why an Update Is Needed

^13^C MFA has been developed rapidly in the last decade. These developments have been impelled, in particular, by advances in analytical measurement technologies where MS and NMR based approaches have been extended in scope and optimized in speed, resolution, precision, and accuracy (Moseley et al., 2011; Choi et al., 2012; Giraudeau et al., 2012; McCloskey et al., 2016a; Nilsson and Jain, 2016; Borkum et al., 2017; Kappelmann et al., 2017; Mairinger and Hann, 2017; Su et al., 2017). In turn, these developments triggered the setup of more comprehensive network models (Gopalakrishnan and Maranas, 2015; McCloskey et al., 2016b; Nilsson and Jain, 2016). Also INST ^13^C MFA application scenarios have become more commonplace (Niedenführ et al., 2015; Cheah and Young, 2018; Delp et al., 2018; Gopalakrishnan et al., 2018). In view of these developments, existing formats have several limitations making a revision necessary.

Two decades of experiences with planning, modeling and analyzing ILEs and the continuous exchange with the 13CFLUX(2) user community have led to the specification of the updated FluxML format, which we present in this work. FluxML now covers isotopically stationary and non-stationary ILEs and is fully universal in terms of network, atom transition, measurement (error), and constraint formulation, including the use of multiple isotopes as tracers. It should be noted that the involved design processes, which we discuss in the following, were driven by the pragmatism to support modelers. Nevertheless, the FluxML format aims at a canonical model representation and follows the recommendations provided by the COMBINE (COmputational Modeling in BIology NEtwork, http://co.mbine.org/) initiative.

### Design Decision 1—Scope: Data Pre-processing Is Not Part of FluxML

Measurement instruments generate raw data that first must be processed to be utilizable for ^13^C MFA. For example, fractional labeling patterns must be extracted from NMR or MS spectra. This includes the identification of target fragments followed by the determination of their abundance by peak integration. For INST ^13^C MFA, in addition, absolute intracellular pool sizes are to be determined. Here, special care has to be taken to correct for known biases in the sampling procedure (e.g., quenching, cell separation, and metabolite extraction). For example, the loss of intracellular metabolites during quenching (known as *leakage effect*) has to be counteracted by application of advanced protocols (Noack and Wiechert, 2014). For both quantities, the labels and pool sizes, standardization and modeling the propagation of the measurement error throughout the analytical processing pipelines is becoming best practice (Tillack et al., 2012; Mairinger et al., 2018).

On the other hand, most software systems for ^13^C MFA emulate metabolite backbones rather than the analytically observed molecules. This means, that the data derived from the raw mass spectra must be corrected for “artificial” and/or “natural” isotope labeling contributions before conforming with ^13^C MFA (Lee et al., 1991; Fernandez et al., 1996; Wahl et al., 2004; Jungreuthmayer et al., 2016; Niedenführ et al., 2016; Su et al., 2017). Also, the specific chemical nature of the analyte mixture and the analysis technique employed might lead to distorted observations, such as proton-loss/gain, which require correction prior to model integration (Poskar et al., 2012). In addition, non-negligible inoculation residues or preliminary labeling sampling times may bias the interpretation of labeling enrichments in the classical case and need, thus, to be corrected (van Winden et al., 2001; Wiechert and Nöh, 2005). Finally, cell-specific external rates and their errors are calculated from cultivation data (concentration time courses of extracellular metabolites, off-gas analysis, biomass composition etc.) by means of simple regression (Murphy and Young, 2013), differentiation after smoothing (Llaneras and Picó, 2007), stochastic filtering (Cinquemani et al., 2017), or tailored bioprocess models (Noack et al., 2011).

That said, it becomes clear that such pre-processing procedures are extremely eclectic and heterogeneous, require a high degree of expertise, and underlie continuous change due to changing experimental setups, instrumentation, vendor formats, and analytical method developments. Recently, the metabolomics community got sensitized about their needs for reporting standards. Data formats and repositories are now under development, to report and store raw data along with its meta-information (Kale et al., 2016; Rocca-Serra et al., 2016). To avoid duplication, FluxML includes only those details about the evaluation procedures that contain the necessary key information about the measurement data that is actually used for producing the flux map (i.e., the *use data*, s. a. Section Experimental Data). The decision to not incorporate data pre-processing is also reasonable from a computer science perspective, since encapsulating complex designs in compact, orthogonal modules limits the overall complexity of the specification and eases future developments.

### Design Decision 2—Technical Considerations: An XML Format for ^13^C MFA

Generally, a modeling language must have a clearly defined syntax and succinct and precise specification of its semantics, for the computer but also for the human if necessary. Re-employing language concepts that are accepted by the target audience help to reduce learning hurdles. With SBML, a XML dialect is already available that is familiar to systems biologists. Technically, the design of FluxML was influenced by SBML as well as the following general considerations:

▪ ^13^C MFA is embedded in workflows consisting of raw data acquisition, customized pre- and post-data evaluation, visualization, computational experimental design and further processes interfacing digital data. Repeated and iterative tool application is commonplace (Dalman et al., 2016). Therefore, it must be possible to use a ^13^C MFA tool on a distributed computing platform, at best as a web service. In this scenario, XML is by far the most ubiquitous information exchange format worldwide. Nowadays, XML formats are commonly used for the structured information exchange in computational biology.
▪ For large-scale networks and complex experimental setups the specific software configuration tasks, i.e., how the computational model is actually created and mapped to the internal data structures of a simulator, are error-prone. For this reason, all required data structures must be generated automatically by some kind of model compiler. For dealing with XML files, hundreds of off-the-shelf parsing, verification and transformation tools are available. This eases the writing of processing software for developers.
▪ Clearly, XML is not designed for a human reader, risking low acceptance among biologists. However, as the SBML success story exemplifies, this argument becomes invalid as soon as convenient, at best graphical, tools for model editing, validation, and formatted export are available. In the scenario of large-scale modeling and proofreading, diagnosis of inconsistencies in the model formulation is vitally important. Using structured XML entails the capability to benefit from powerful validation mechanisms that allow for the precise diagnosis of syntactical and semantical errors.
▪ XML combines the flexibility of full configurability with the user-friendliness of lowering complexity. It is very easy to extract partial information from XML files or to extend XML formats with additional information. With this, XML allows model pre-configuration to present only those parts to the experimentalist that are relevant, e.g., because they change over a series of experiments.
▪ A frequently discussed issue in simulation technology is the separation of model structures, model parameters, and measurement data. For instance, it is desirable to use the same model structure for identically configured ILEs, with parameters and data changed. Using XML, this poses no problem as long as model, parameters, and data are deposited in different branches of the XML tree. Furthermore, XML provides mechanisms to store associated model structures and data in separate files.

### Design Decision 3—FluxML a Domain-Specific Language

One of the key design objectives of FluxML was to allow for automated model interpretation (analysis and code generation) for large-scale isotope labeling networks without forcing the modeler to resort to text-based specifications of low-level model description languages. Here, it could be argued that the flexibility of general description languages like CellML, offering a low-level description of the mathematical equations, is unraveled when new experimental or analytical paradigms become available. However, the generality comes at the price of readability and clearly challenges the proofreading capabilities of the modeler.

On the other hand, isotope labeling networks share many aspects with stoichiometric metabolic network models. For this reason, FluxML and SBML have a common subset of information that contains the metabolite and reaction names as well as the network stoichiometry and flux constraints. While reaction kinetic information is currently not in the scope of ^13^C MFA, atom transitions, tracer mixtures, as well as experimental data are not part of SBML. Thus, the set of common features is not that large. Recently, an attempt has been made to encode the surplus information required for ^13^C MFA in the SBML notation (Birkel et al., 2017). Here, the construct *notes* (extending *reaction* and *species* in the notion of SBML) has been utilized to express carbon atom mappings and measurement data. However, because atom transitions and measurement specifications are vital for generating the essential mathematical system (Weitzel et al., 2007), it is clear that specifying this information in optional add-on elements, such as *notes*, complicates validation and consistency checking enormously. Hence, such a solution is not recommended by the SBML designers^2^.

Taken together, these reasons speak in favor of the domain-specific standalone XML-based language. We followed the example of SBML and adopted those parts belonging to the common language subset with only minor changes to FluxML. The common subset is then extended by the information necessary to specify ILEs. Firstly, this way the entry level for a newcomer already familiar with SBML is lowered. Secondly, extracting the common information from a FluxML file and generating a rudimentary SBML document, or vice versa, is fairly straightforward.

## FluxML in a Nutshell

FluxML development branches are organized in major *Levels* and minor *Versions*. Level 1 is dedicated to isotopically stationary ^13^C MFA (Weitzel et al., 2013) while Level 2 covers both, the isotopically stationary and non-stationary cases. During language design special care has been taken to keep Level 2 backward compatible to Level 1, meaning that existing simulation tools designed for using the published FluxML version (Weitzel et al., 2013) do not need adaption when being used with Level 2 files. This helps third-party software developers using Level 1-models as input in keeping their versions stable. Lastly, FluxML Level 3 has been developed which extends Level 2 to the general case of multiple isotopically labeled elements. Here, for obvious reasons, backward compatibility could no longer be maintained.

**Figure 2:**
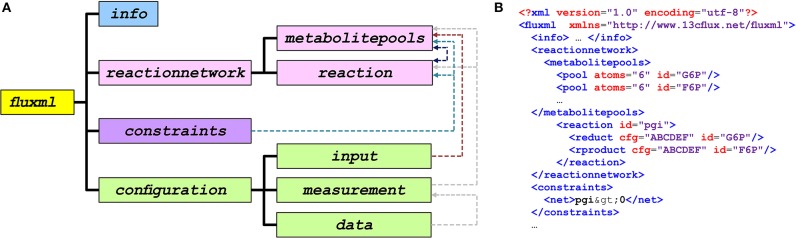
Structure of FluxML. **(A)** Overall hierarchical structure of FluxML. The top-level element *fluxml* contains a number of child elements. Dashed lines indicate relationships between elements. A class diagram showing FluxML data structures in the Unified Modeling Language UML (ISO/IEC 19505) are provided in Supplementary S1 Figure 2.1. **(B)** Excerpt of a FluxML document.

The general hierarchical structure of FluxML documents that are common for all Levels is shown in Figure 2. Figure 2A overviews the main elements of the FluxML language while Figure 2B shows a code excerpt from the serialization of a model. The top-level element *fluxml* contains the elements *info*, for providing basic information about the model, and *reactionnetwork* containing metabolites and reactions which, together with the *constraints* element define the isotope network structure. An important key concept of FluxML, which is not present in SBML, is that of *configurations*. *configurations* entail the convenient possibility to connect the same model structure with different experimental or simulation settings. In this way, instances that, for example, differ in the selection of the tracer mixture, flux parametrization, and/or measurement configuration can be stored in different *configurations* sections within one model file. Another core concept of FluxML, distinguishing it from SBML, is the incorporation of experimental data. Here, the measurement data declaration is separated from the data specification by the *measurement* sub-elements *model* and *data*, respectively. Finally, the *simulation* element contains details about the model parameterizations in terms of free model parameters as well as their values.

Instead of an exhaustive language description, only the major features of each FluxML section should be highlighted in the following, in particular those that eliminate limitations of the FTBL format and represent novel developments in the field.

### Metabolites, Reaction Network Structure, and Atom Mappings

Clearly, biochemical reaction steps and their atom transitions constitute the core of any ^13^C MFA model. The section *reactionnetwork* defines the metabolite pools, the reactions interconnecting them, and atom transitions which, altogether, give rise to the network structure of the ^13^C MFA model. Each metabolite and reaction is labeled with a unique identifier (*id*) which assures its consistent usage throughout the FluxML document (cf. Figure 2B). Here, the atom enumerations are of particular importance not only for tracking the atoms, but also the correct association of the measured labeling fractions with the reactants.

Before going into specification details, it is appropriate to briefly summarize the most important facts about the network and atom transition compilation. Although there are plenty of ways to retrieve information from reaction databases (KEGG [http://www.genome.jp/kegg/], BioCyc [https://biocyc.org/], MetRxn Kumar et al., 2012), fluxomics collections (Zhang et al., 2014) [http://www.cecafdb.org], model repositories such as Biomodels [https://www.ebi.ac.uk/biomodels-main/] and BIGG [http://bigg.ucsd.edu/], as well as algorithmic approaches (Kumar and Maranas, 2014; Hadadi et al., 2017), there is currently no “one” curated source containing all the structural information needed for setting up a ^13^C MFA model. In this context it is worthwhile to remember that solid biochemical knowledge beyond simple net reaction stoichiometry is needed. One prominent example is the transketolase- and transaldolase-catalyzed reaction complex in the pentose phosphate pathway (PPP) where the kinetic enzyme mechanism impacts the formulation of the associated carbon atom transitions (van Winden et al., 2001; Kleijn et al., 2005). Considering this, it is fallacious to solely rely on information available in biochemistry textbooks and reaction databases. Further study-specific factors to be considered are reaction reversibilities, transamination reactions, isoenzymes showing evidence for differences in substrate affinity and activity, and (micro-)compartmentalization due to metabolite channeling or metabolically inactive pools (van Winden et al., 2001). All these factors may influence flux inference from available labeling distributions. On the other hand, it is common to simplify reaction networks, e.g., by lumping “linear” reaction chains into one surrogate reaction when the labeling distribution (and incorporation speed in case of INST) is not affected.

The mentioned considerations imply that the ^13^C MFA model compilation procedure is hardly automatable, at least for non-standard cases. Currently the best way to build and verify the network model from scratch is to use various information sources and a visual tool for specification and proofreading purposes (Nöh et al., 2015). Having a list of relevant reactions and metabolites at hand, different naming conventions exist for representing the associated atom transitions. Traditionally, case-sensitive characters have been used to specify carbon transitions, as exemplified by the Fructose-bisphosphate aldolase reaction in glycolysis (in biochemical enumeration and FTBL notation):


emp4: FBP > GAP + DHAP
   #abcdef > #cba + #def


Although this notation is convenient for an end-user, and still used by many software tools, it obviously does not fulfill the aforementioned requirements of a universal language. For this reason, atom transitions are specified in FluxML as follows:

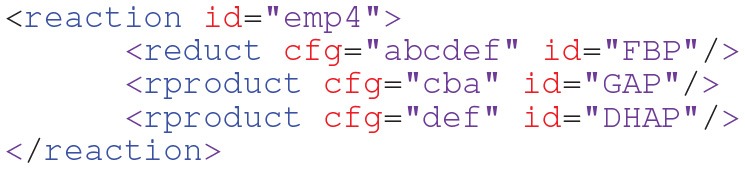



**Figure 3:**
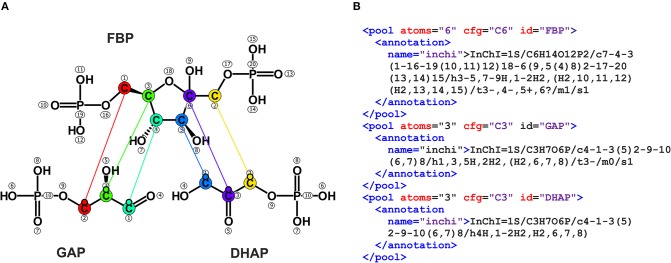
Fructose-bisphosphate aldolase reaction *emp4*. **(A)** Carbon atom transitions. Atoms are enumerated according to their appearance in the InChI string. Off-the-shelf chemistry programs provide visualization of the molecule structures and atom numbers. Specialized tools, such as the Omix visualization software, allows for visual specification of atom transitions as well as the export of the results as in FluxML, releasing the user from any peculiar enumeration issue (Nöh et al., 2015). **(B)** Metabolite specifications in FluxML format annotated with InChI strings. The *cfg* argument reports the atomic elements involved in the transition network (C6 for the six carbon atoms of *F6P*), while the InChI string implicitly contains the enumeration order of the atoms. Once specified in the *reactionnetwork* section of the FluxML tree, *atoms* and *cfg* specifications for the metabolite with the name id are binding for the whole document.

This way, a reaction (*reaction*) can accommodate an arbitrary number of educts (*reduct*) and products (*rproduct*). These refer to unique metabolite names that are declared in the *metabolitepools* section of the FluxML document along with the definition of label-carrying atom types and numbers (cf. Figure 3B).

An implicit assumption underlying both *emp4* representations is the use of the IUPAC recommendation for coding the carbon atom-character relation of the metabolites. Herein, the lettering starts with the highest oxidized group of a molecule following the main carbon chain etc. For instance, following the biochemical enumeration the first carbon atom of glyceraldehyde 3-phosphate (GAP) is the one in proximity of the phosphate group (cf. Figure 3A). Due to its popularity among biochemists the IUPAC “biochemical enumeration scheme” has settled as pseudo-standard.

However, having genome-sized networks and multi-element ILEs in mind, this enumeration practice becomes questionable. In this situation, a veritable alternative is the International Chemical Identifier (InChI) (Heller et al., 2015). The InChI identifier is a computer-generated unique character string for encoding molecular structures that is widely accepted in the chemical community. The InChI identifier does not only facilitate database/web-search and information exchange in the field of metabolomics, it also comes with an outstanding merit for ^13^C MFA model exchange: InChI gives an identifier and canonical ordering to each atom of a metabolite (except for hydrogen). Thereby, employing InChI strings for metabolite declaration and atom enumeration makes network descriptions self-contained and exchangeable.

As an example, Figure 3 shows the atom numbering as provided by the InChI software [http://www.inchi-trust.org/]. Accordingly, the carbon atom transitions for the aldolase reaction *emp4* in FluxML notation is:

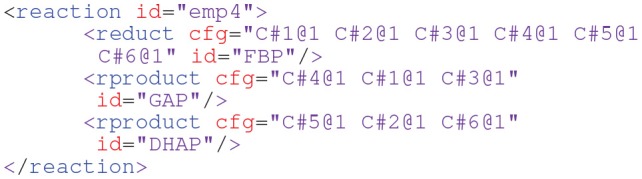



Herein, the atoms of the educt *FBP* are represented by white-space separated list of entries of the form *element#canonical_atom_index@educt_index* which are mapped to the respective atom positions in the products. Of course, the mapping can still be expressed by letters^3^. However, the use of the more complicated *#@* notation pays off immediately when ILEs with multiple isotopic tracers are considered. Using the InChI notation, generalization of transitions is straightforward, without losing readability, as exemplified with the glutamate dehydrogenase *gdhA* converting α-ketoglutarate (*AKG*) and ammonia (*NH3*) to L-glutamate (*GLU*):

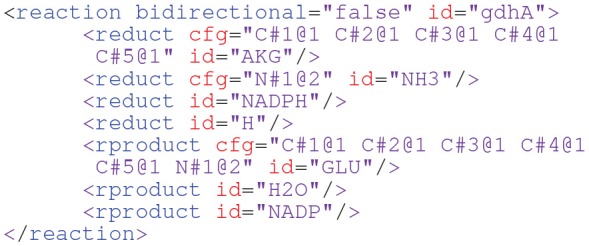



Herein co-factors *NADPH, NADP, H*, and *H2O* (i.e., metabolites that do not carry labeled material in the scope of the model) are explicitly specified as reaction partners, a feature that helps to keep FluxML and SBML reaction network representations consistent.

### Stoichiometric Constraints

Constraints on the fluxes that impose bounds on the reaction rates on top of the stoichiometric mass balances are important components of any flux model. Typically, such constraints express principled condition-dependent biological or simulation settings. Unfortunately, these equality or inequality relations remain undocumented in ^13^C MFA publications and are, in our experience, a frequent reason why the reproduction of published flux maps fails. Hence, it is vitally important to bundle the complete constraint set together with the model.

An aspect which is conceptually closely related to flux constraints is that of reaction directionality. Here, it often depends on the actual *in vivo* conditions whether a reversible reaction operates in forward and backward direction (*bidirectional*) or only in one of the directions (*unidirectional*). In ^13^C MFA this setting must be carefully considered since it impacts flux inferences. Technically, in purely stoichiometric models bidirectional reactions are split into non-negative forward and backward parts. In ^13^C MFA, however, it is common to use an alternative description for bidirectional reactions, i.e., that of net and exchange fluxes (Wiechert and de Graaf, 1997). Exchange fluxes are net-neutral intracellular material exchanges between reactants (not to be confused with extracellular rates). An advantage of the net/exchange flux system over the backward/forward formulation is that it leads to a “decoupling” of the underlying mathematical equation system for the two flux types, making it easier to express assumptions on both of them.

In FluxML, reaction directionalities are set with the Boolean attribute *bidirectional* = "true" or *bidirectional="false"* (cf. *gdhA* reaction above). Since net fluxes can take positive and negative values (n.b., exchange fluxes are always non-negative), typical assumptions on net fluxes are “sign” constraints (e.g., *v*^*net*^ ≥ 0) indicating known net flux directions owing to thermodynamic reasoning, upper limits to individual fluxes from enzyme capacity measurements ($v^{net}\leq v_{\max}^{net}$), or specific flux ranges ($v_{\min}^{net}\leq v^{net}\leq v_{\max}^{net}$). Similarly, upper boundaries for exchange fluxes may be applicable for thermodynamic (Wiechert, 2007) or numerical reasons (Theorell et al., 2017). Finally, net and exchange fluxes, respectively, can be related through equality and inequality relations to express further specific relationships such as the rate equalities of scrambling reactions (cf. Section Symmetric (Scrambling) Reactions). The following excerpt gives a typical example:

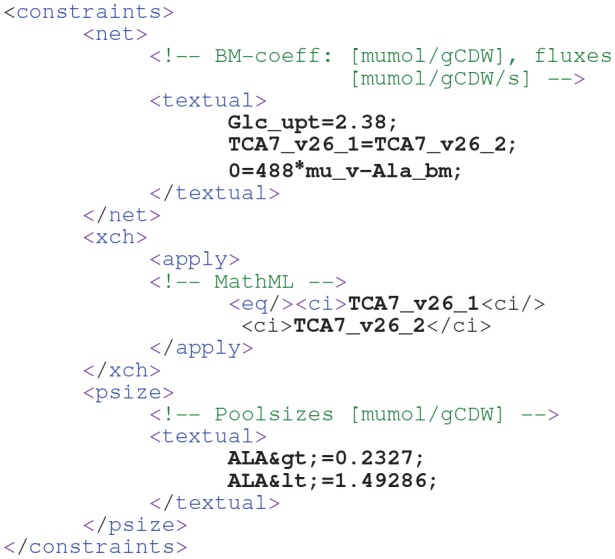



Here the glucose uptake rate (*Glc_upt*) is assigned to a value of 2.38 [μmol/g_CDW_/s] and net as well as exchange fluxes of the two succinate dehydrogenase reaction variants (*TCA7_v26_1,2*) converting succinate to fumarate are equalized. The third entry encodes a biomass efflux (*Ala_bm*) that is proportional to the cell growth flux (*mu_v*). Importantly, mathematical relations can be expressed in human-friendly text-string representation as well as in Content-MathML [https://www.w3.org/TR/MathML3/chapter4.html] (cf. Supplementary S1 Section 4.1 for an example). Besides the fluxes, pool sizes may also be subject to restrictions. For alanine (*ALA*) a lower and upper boundary is specified, indicated by the XML entities *&gt;* (>) and *&lt;* (<), respectively.

### Symmetric (Scrambling) Reactions

**Figure 4:**
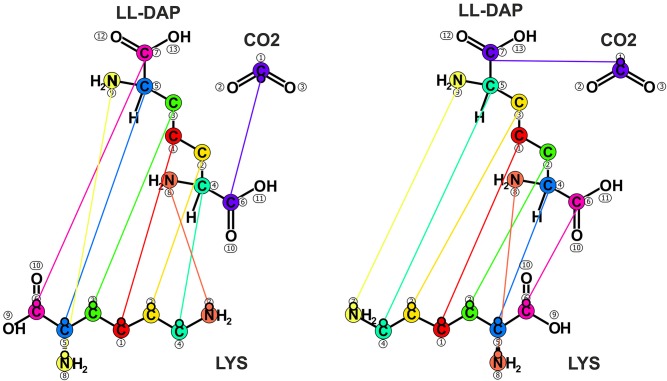
Scrambling reaction. Carbon-nitrogen mapping variants for the *diaminopimelate decarboxylase* reaction catalyzing the decarboxylation of LL-2,6-diaminopimelate (*LL-DAP*) to L-lysine (*LYS*) and *CO2*. Due to its rotation symmetry, *LL-DAP* has two biochemically indistinguishable carboxyl groups, resulting in two different mapping variants.

Scrambling reactions constitute a special class of reactions that involve symmetric molecules, i.e., molecules that are biochemically indistinguishable due to their rotational symmetry. For instance, the metabolite LL-2,6-diaminopimelate (*LL-DAP*), an intermediate of the lysine biosynthesis pathway, contains a rotation axis which gives two symmetric groups (cf. Figure 4). In the general case of *n* symmetric groups, *n*! different mapping variants exist, which all have to be specified to describe the emerging labeling patterns correctly.

Technically, any scrambling reaction can be specified as a set of reaction variants, implementing the alternative atom mappings. Here, it is typically assumed that the catalyzing enzyme treats all biochemically indistinguishable isotopomers equally, resulting in identical fluxes of each of the mapping variants. In turn, the associated fluxes are set equal by formulating appropriate equality constraints. Depending on the symmetry level this approach can lead to numerous “virtual” reactions that have to be handled appropriately, also in the post-processing of the results, e.g., the visualization of the flux map. To alleviate the specification process, specific elements (*variant*) and attributes (*ratio*) for modeling scrambling reactions have been introduced to FluxML. The following listing showcases the specification the *diaminopimelate decarboxylase* scrambling reaction *AA13_v49* by means of the variant notation (cf. Figure 4 and Supplementary S1 Section 4.2 for the traditional specification):

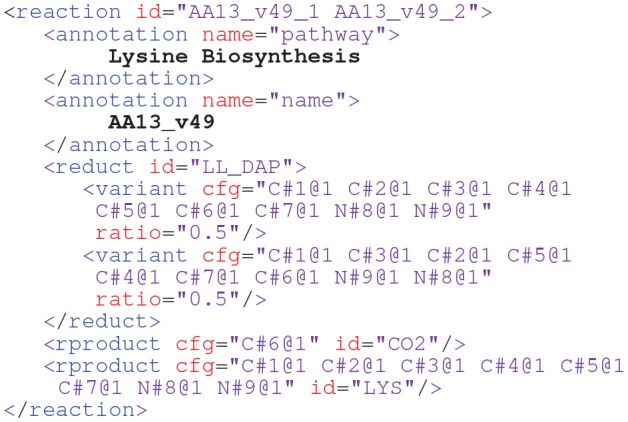



Herein, two reaction variants *AA13_v49_1* and *AA13_v49_2* are specified, induced by the symmetry of the educt *LL_DAP*, having fixed equal fluxes (*ratio* = *"0.5"*). Furthermore, the FluxML excerpt shows how elements can be enriched with additional information, e.g., associating the reaction variants to their superordinate reaction (*AA13_v49*) and the pathway names.

### Configurations

Experience shows that after an initial set-up phase, ^13^C MFA evaluation workflows are accompanied by a series of minor model modifications. Here, the majority of differences lie in the settings of constraints, parameter sets and values, and the composition of data sets, while the model structure itself remains largely untouched. Configurations are created having these experiences in mind. In a *configuration* branch of the FluxML tree, *input*-, *constraints*-, *measurement*-, and simulation-settings are bundled, each specific to one ILE or simulation experiment. A FluxML document can then contain an arbitrary number of such configurations.

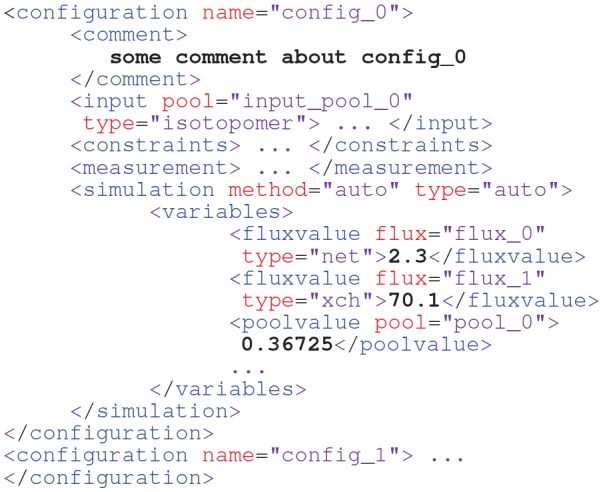



Combined with the reaction network, each single configuration constitutes a complete ^13^C MFA model. Consequently, the use of configurations releases the modeler from the necessity to duplicate files beyond necessity and, thus, makes model management more transparent and less error-prone. A typical application scenario where this is enormously useful, are so called parallel ILEs (cf. Section Parallel Labeling Experiments for a worked example). Therewith, configurations are one of the most powerful paradigms of FluxML, as compared to its predecessor FTBL and other modeling languages such as SBML. In the following, the single *configuration* elements are briefly overviewed.

### Input Mixture Specification

A broad variety of labeled substrates has been used in ^13^C MFA, individually or in mixtures, to elucidate metabolic fluxes (Crown et al., 2015; Nöh et al., 2018). Optimal experimental design (OED) heuristics give guidance on the selection of the tracer mixture to maximize the chance of the ILE to be informative about the fluxes. How to select the labeled species for a specific question under study, rather than taking a standard experimental design, is a computational question par excellence (see Section Special Settings for ILE Design). As such, the composition of the substrate pools in terms of labeled species has been subject of various design studies and the OED of ILEs has become a built-in feature of contemporary software systems.

In FluxML, the composition of a substrate labeling is specified in the input section by supplying the fractions of the input species present in the substrate pool(s), usually in form of isotopomers. Here, it must be taken into account that neither “unlabeled” nor “labeled” proportions are 100% pure in practice: the abundance of ^12^C and ^13^C isotopes (0.9893 and 0.0107, respectively) leads to a natural variation in the isotopomer compositions. In case of naturally labeled substrates, it is sufficient to correct for the variation in each single atom position while neglecting occurrences of combinations of two or more labeled positions (the error due to the occurrence of multiple labeled molecules is below 1.1·10^−4^ and decreases rapidly with increasing number of labeled positions). As an example, the formulation for [^12^C] glucose is:

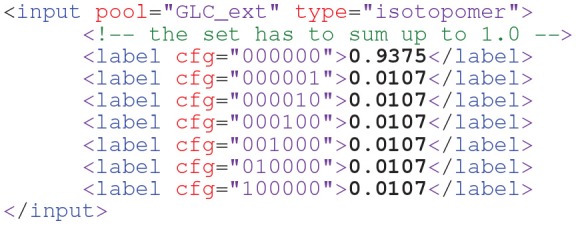



Commercially available isotopic tracers vary in their isotopic purity in a cost-dependent manner, implying that not only the natural abundance impacts the fractions of the single labeled species, but also the manufacturing and purification quality. In FluxML, the attributes *purity* and *costs* have been created to precisely express these contributions. As an example, a glucose mixture consisting of 77% [1-^13^C]-, 20.5% [U-^13^C]-, and 2.5% [^12^C]-glucose is specified in the following succinct way:

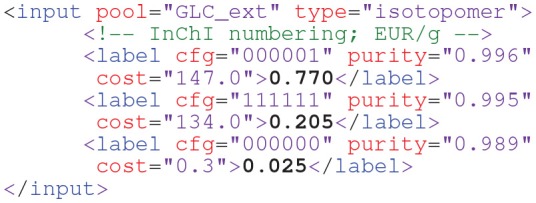



The extension to the multiple-element input substrate specification is then straightforward (cf. Supplementary S1 Section 4.3).

For designing ILEs, different substrate sources are mixed with the aim to determine those tracer proportions that are optimally informative about the fluxes. Arbitrary mixtures of labeled substrates are modeled in FluxML by specifying one uptake flux per tracer in the metabolic network. All these uptake fluxes then amount to the total uptake rate of the corresponding substrate which is specified in the constraint section of a FluxML document, for example:

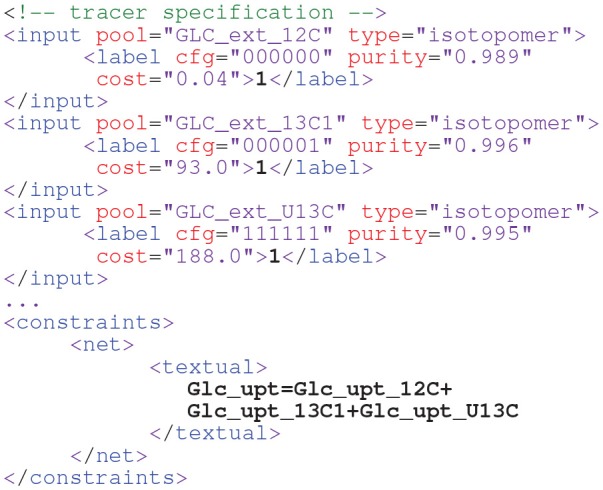



where *Glc_upt* is the total uptake rate and *Glc_upt_12C, Glc_upt_13C1, Glc_upt_U13C* are the individual uptake rates of naturally [^12^C]-, [1-^13^C]-, and fully [U-^13^C]-labeled glucoses, respectively. Uptake fluxes are canonically unidirectional, usually with an extracellular rate assigned (cf. Section Extracellular Rates). For the case that intra- and extracellular metabolites are exchanged, a specification example is given in Supplementary S1 Section 4.4.

**Figure 5:**
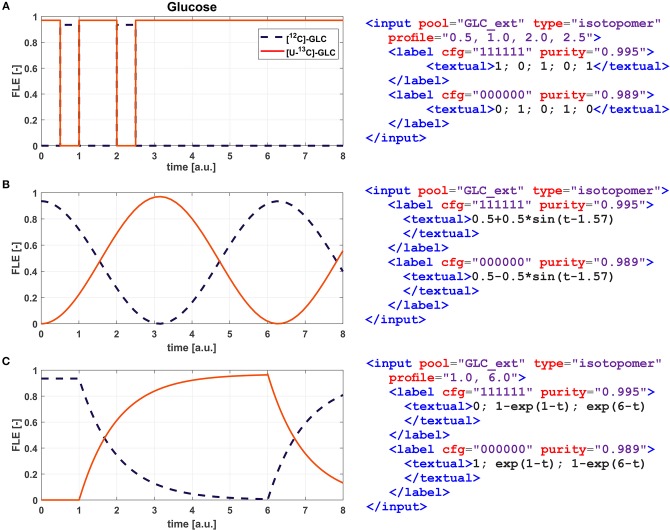
Isotopic substrate labeling profiles. Showcased are a simple repetitive switch between fully (red solid line) and naturally labeled glucose (blue dashed line) specified via a Boolean condition for each time interval **(A)**, a sinus with wavelength 2π **(B)**, and an exponential enrichment-decay curve **(C)**. Shown are fractional labeling enrichments (FLE). The species of each input pool must sum up to 1.0 to define a valid profile. Contributions attributed to impurities are not displayed in the charts.

Principally, the labeling states of the intracellular metabolites depend on the input labeling composition which is usually constantly administered. For INST ILEs such kind of restriction is no longer mandatory, therewith paving the way for the targeted exploitation of dynamic labeling profiles to design highly informative ILEs. Certainly, the most simplistic form of labeling profiles is a repetitive switch between two isotopomer species of a substrate. But also more sophisticated profiles, such as sinusoidal and pulse-width modulated waveforms, have been considered theoretically (Sokol and Portais, 2015). Another scenario, where profiles are of practical value, is when ILEs are conducted under cultivation conditions where the administered carbon source is present in excess. In FluxML, such labeling profile functions can be flexibly specified (cf. Figure 5).

### Specific Constraints

Besides constraints that are inherently linked to the network structure irrespective of the experimental conditions (i.e., globally valid constraints, cf. Section Stoichiometry Constraints), FluxML configurations allow to specify additional *specific* constraints, i.e., those that may only be valid in the context of a concrete experimental setting. For instance, the flux solution space can be tightened by such specific constraints in the context of simulation experiments. Both types of constraints are syntactically equivalent.

### Experimental Data

Measurements are an integral part of ^13^C MFA models, being the basis of flux inference. But also, ^13^C MFA codes are tuned for specific measurement types (mostly MS, cf. Supplementary S1 Table 1.1). The reason is that the labeling system that is actually needed to describe the sub-set of observable labeling states can be tremendously smaller than the labeling network describing all intracellular labeling states. For the reduction of the high-dimensional labeling systems powerful graph theoretic algorithms have been developed (Weitzel et al., 2007), which are implemented in the high-performance code 13CFLUX2. Consequently, the resulting reduced labeling systems intimately rely on the specific measurement configuration. Notably, the reduction crucially impacts the computational efficiency of flux fitting, rather than the final flux map. Before explaining how the specific measurement setup of an ILE is specified in FluxML, some general remarks on the present measurement equipment are appropriate.

#### Modeling Data

Measurement models provide a link between the models' state variables and parameters (fluxes, pool sizes in the case of INST) and the observables (extracellular rates, pool sizes, labeling measurements). These three data models are essentially linear, which is trivial to see for the first two types. Therefore, we concentrate on the modeling of the labeling patterns. Consider a metabolite fragment *M* with *n* atom positions. Each atom can be present in one of *k* labeling states ({0,1} for ^12^C, ^13^C, and ^14^N, ^15^N, {0,1,2} for ^16^O, ^17^O, ^18^O etc.). For the isotopomer fractions of *M* then it holds:

(1) $$\begin{array}{r} 0\leq m_{k_{1},k_{2},\ldots ,k_{n}}\leq 1.0\;\text{with}\underset{k_{1},k_{2},\ldots ,k_{n}}{\sum }m_{k_{1},k_{2},\ldots ,k_{n}}=1.0 \end{array}$$

With the isotopomer fractions any labeling measurement is formulated based on the following criteria, which should be obeyed by any well-calibrated measurement procedure:

▪ Each single isotopomer of *M* contributes to the spectrum (including a zero contribution).
▪ All isotopomer contributions superpose linearly.
▪ The signal intensities scale proportionally with the total amount of the specific isotopomer in a sample.
▪ This superposition of contributions results in number of distinguishable peaks.
▪ These peaks can be properly identified.
▪ Signal intensities are quantified, usually by integrating the respective peak areas.

To make these considerations more concrete, the case of a mass isotopomer distribution (MID) generated in MS is discussed. The MID of an analyte is the vector of fractional labeling enrichments that are derived from the contribution of the single peak areas relative to the sum of all peak areas of the respective analyte. Apart from aspects of pre-processing (cf. Section Design Decision 1—Scope: Data Pre-processing Is Not Part of FluxML), an ideal MS ion chromatogram of a metabolite fragment *M* with three carbon atoms, contains four distinguishable peaks (m.0, m.1, m.2, m.3) to which in total 2^3^ = 8 isotopomers contribute. Precisely, the M000 isotopomer contributes to the m.0, M001, M010, M100 isotopomers to the m.1, M011, M101, M110 isotopomers to the m.2, and the M111 isotopomer to the m.3 peak, respectively. The relation between isotopomers (**x**_*M*_) and the MID of *M* (**y**_*M*_) can be represented using matrix notation:

(2) $$\underset{y_{M}}{\underset{︸}{\left(\begin{array}{c} y_{M}^{m.0}\\ y_{M}^{m.1}\\ y_{M}^{m.2}\\ y_{M}^{m.3} \end{array}\right)}}=\underset{M_{M}}{\underset{︸}{\left(\begin{array}{cccccccc} 1 & 0 & 0 & 0 & 0 & 0 & 0 & 0\\ 0 & 1 & 1 & 0 & 1 & 0 & 0 & 0\\ 0 & 0 & 0 & 1 & 0 & 1 & 1 & 0\\ 0 & 0 & 0 & 0 & 0 & 0 & 0 & 1 \end{array}\right)}}\;\cdot \;\underset{x_{M}}{\underset{︸}{\left(\begin{array}{c} m_{000}\\ m_{001}\\ m_{010}\\ m_{011}\\ m_{100}\\ m_{101}\\ m_{110}\\ m_{111} \end{array}\right)}}$$

with **M**_*M*_ the MS *measurement matrix* of metabolite fragment *M*. In principle, this measurement matrix scheme holds true for other analytical techniques (with different sparsity pattern of the measurement matrix **M**_*M*_), as long as the measurements obey the common criteria of good analytical practice described before. Because in Equation (2) appropriate re-scaling of the intensities by (unknown) group-specific scaling factors ω_*M*_ may be required to match the simulated enrichments (Wiechert et al., 1999), the general *measurement models* read:

(3) $$y_{M}=\omega_{M}\;\cdot \;M_{M}\;\cdot \;x_{M},y_{M}\left(t_{i}\right)=\omega_{M,\;t_{i}}\;\cdot \;M_{M,\;t_{i}}\;\cdot \;x_{M}\left(t_{i}\right)$$

for the isotopically stationary and non-stationary cases, respectively. It should be remarked that isotopomer fractions are not the only systematic that can be used for expressing labeling states. Alternatives are cumomers (Möllney et al., 1999), EMUs (Antoniewicz et al., 2007), or tandemers (Tepper and Shlomi, 2015). Since all three labeling systematics can be linearly transformed into isotopomer fractions, the general measurement model formulations given in Equation (3) are equally valid for these alternate frameworks.

#### Measurement Specification in FluxML

Experimental data are located in the *measurement* branch of the FluxML tree. By design, we distinguish between the *declaration* of the measurements (< *model*>) and the *specification* of the quantitative data (< *data*>):

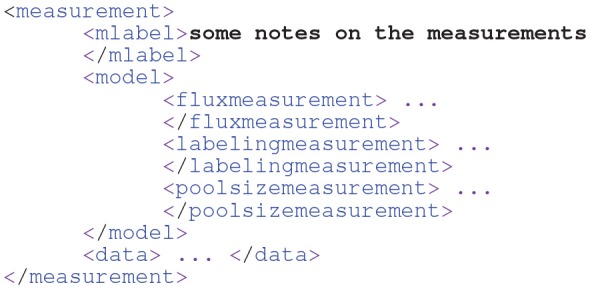



As in the case of reactions and metabolite pools, each measurement group must be accompanied with a unique identifier (*id*) to unambiguously crosslink the declared reactions and metabolite pools with the specified measured entities (cf. Figure 2).

##### Extracellular rates (< ***fluxmeasurement***> section, Level 1+)

Flux measurements are essential to any network-wide ^13^C MFA study. Uptake and secretion fluxes are net rates, specified one-by-one with the following notation:

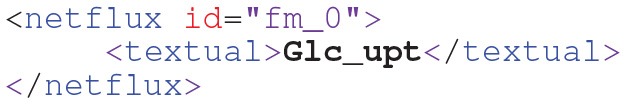



On the other hand, FluxML also allows for the formulation of functional relations between model parameters and to equip these with measurements. This feature can be used to incorporate flux ratios, e.g., obtained using FiatFlux or SUMOFLUX (Kogadeeva and Zamboni, 2016):

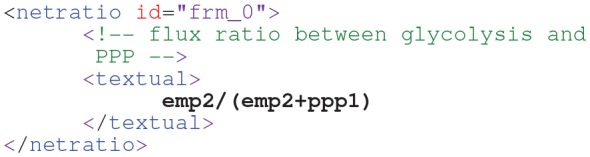



##### Isotopic labeling (< labelingmeasurement> section, Level 1+)

The remarks on measurement models above make clear that in practice only one approach works for a universal specification language: The user should be enabled to compose specific measurement configurations from predefined basic expressions (*primitives*) with which more complex measurement specifications can be expressed. These primitives describe (real or envisioned) measurements with concise code fragments. Consequently, in FluxML labeling spectra are composed by linear combinations of measured signals:

1. The most basic primitive specifies a single isotopomer fraction:
  *M#010*
  This means that the isotopomer M010, which carries a labeled atom only at its second atom position, contributes to the measurement matrix. As an extension of the isotopomer notation, a positional atom entry can be marked by an “*x*” expressing that no information is available for this position or, with other words, any labeling state is allowed. For example,
  *M#01x*
  denotes the set of isotopomers {M010, M011} (if the third atom position of *M* codes for an element with two possible isotopic labeling states). In terms of the measurement models Equation (3) this means that all isotopomers of the set contribute a “*1*” to the row of the measurement matrix while all other isotopomers lead to a zero entry in **M**_*M*_. If only the symbols “*1*” and “*x*” are used, the notation coincides with the cumomer notation (Wiechert et al., 1999). Labeling patterns of fragments are identified by the associated atom numbers given in squared brackets, e.g. *M[1-2]#*. This way, the seven EMUs (moieties comprising any distinct subset of the compound's atoms Antoniewicz et al., 2007) of *M* are represented by *M[1]#, M[2]#*, *M[3]#, M[1-2]#, M[1,3]#, M[2-3]#,* and *M[1-3]#* (or *M*#)
2. Apart from these primitives, FluxML contains convenient short-notations for expressing measured signals for a plethora of measurement techniques:
  - One-dimensional ^1^H-NMR generate positional enrichment information: 
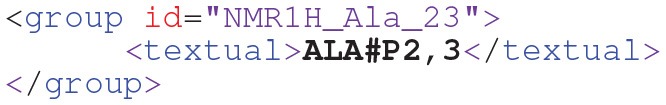
    Here, the positions *P* = 2 and 3 of the metabolite Alanine (*ALA*) are specified, coding for isotopomer fractions that are ^13^C labeled at position *P*. Since the two sets of positional isotopomers interfere, they are combined to one measurement group, named *NMR1H_Ala_23*.
  - Beyond positional observations, two-dimensional ^13^C-NMR can discriminate between certain labeling positions in the direct neighborhood of a ^13^C-labeled position, giving rise to multiplets: peak singlets (*S*) occur, when the focused position is surrounded by unlabeled atoms. Right or left doublets (*DR*, *DL*) emerge if exactly one of the adjacent carbon atoms is labeled with ^13^C. If two surrounding positions are occupied with ^13^C isotopes, double doublets (*DD*) or triplets (*T*) may be obtained. In the following FluxML snippet, two measurement groups of *ALA* are listed, targeting the second and third carbon position, respectively: 
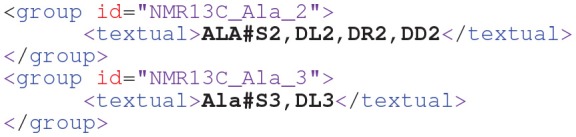
  - In MS measurements all isotopomers with the same number of labeled atoms are pooled, resulting in MIDs as exemplified for the C9 metabolite phenylalanine (*PHE*): 
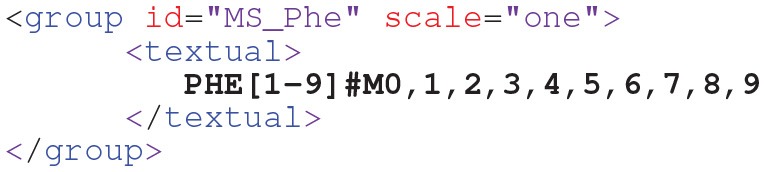
    This measurement group specifies 10 mass isotopomers (m.0,…, m.9) which share a common scaling factorω, as represented by the scale attribute (Möllney et al., 1999). The scale factor is a nuisance parameter that translates between the simulated ([0,1]) and measured ranges of the enrichment data (cf. Equation (3)). This attribute is either *one*, all measurement values of the *PHE* measurement group are taken as specified, or *auto*, meaning that the scale factor is to be determined within the fitting procedure. With FluxML Level 3, also MS data from multiple-isotope tracer experiments can be conveniently specified (cf. Supplementary S1 Section 4.5 for an example).
  - Beyond simple MS, tandem MS has proved to be very informative about fluxes, since it can deliver positional information. In FluxML, tandem MS measurements of *PHE* are specified as follows: 
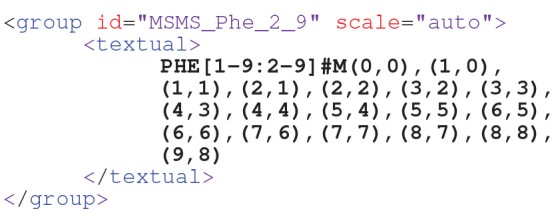
    Here, the first atom range (*1-9*) refers to the precursor ion, whereas the second range (*2-9*) relates to the product ion (i.e., the first carbon atom is filtered). The tuples specify the tandem mass isotopomers defined by the precursor and product ion, respectively.

**Figure 6:**
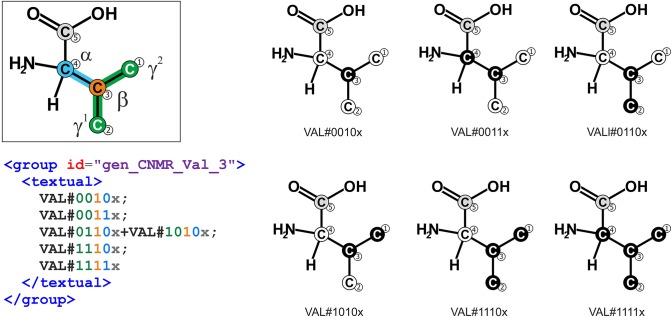
Hypothetical ^13^C-NMR measurement of the amino acid valine (*VAL*). The multiplet of valine-β is given in generalized isotopomer notation. Numbers indicate positions of carbon atoms in InChI enumeration. The labeling states of the first and second carbon atom (γ^1^, γ^2^) are indistinguishable by ^13^C NMR, leading to the sum signal *VAL#0110x*+*VAL#1010x*. In this case, no information about the fifth carbon position is available (gray).

Aside from such shortcuts to specify measurement configurations, the FluxML notation is fully universal because any possible linear measurement combination can be described. This way, arbitrary setups can be expressed, for instance, a ^13^C-NMR measurement of valine (*VAL*, cf. Figure 6). The flexibility of the FluxML syntax is further demonstrated with the formulation of the *summed fractional labeling*, the sum of the fractional labeling of the atoms contained in a molecule (fragment) (Christensen et al., 2002). The summed fractional labeling can be specified by either using the generalized isotopomer notation:

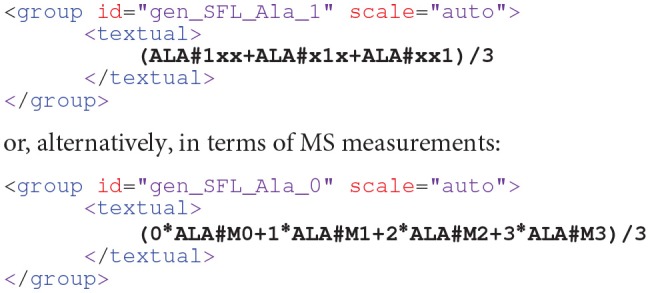



*The isotopically non-stationary case (Level 2*+*):* In contrast to classical, isotopically stationary ^13^C MFA where labeling data sets consist of one single labeling measurement vector, in the INST case labeling measurements are time series data. In FluxML, with Level 2 upwards, the measurement time points are introduced as attributes of the measurement groups. This way, time resolved MIDs of *ALA* can be formulated as follows:

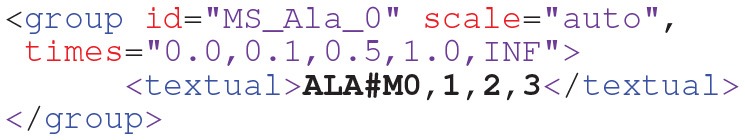



expressing that MIDs of *ALA* are available at five time points (*0.0, 0.1*, *0.5,1.0*,∞). This notation enables joining isotopically stationary and non-stationary data in a single measurement group.

A consideration, which becomes especially important in the INST case, is that fluxes, pool sizes, and time need to be formulated in a coherent physical unit system to produce meaningful results^4^. Metabolic fluxes (and extracellular rates) are amounts of substance transported per time unit. Practically, this means that with the choice of the flux unit, the units of the pool sizes and time are implicitly determined as well. Fluxes are reported in diverse units, e.g., [mmol/g_CDW_/h], [mmol/L_Cell_/s], or [nmol/10^6^ cells/h]. Due to this variety, FluxML does not enforce a specific unit system. However, modelers are strongly advised to document the applied units in the FluxML elements *fluxunit*, *poolsizeunit*, and *timeunit*.

##### Pool sizes (<poolsizemeasurement> section, Level 2+)

Likewise important for flux inference in the INST case are intercellular pool size data. In FluxML they are specified as follows:

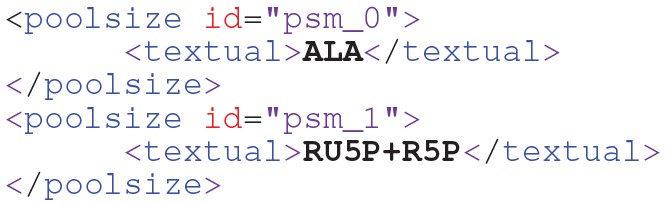



for single and pooled measurements, respectively. Due to the metabolic steady state, pool sizes and extracellular rates remain constant throughout the ILE. Thus, specification of one measurement per entity is appropriate in both cases.

#### Specification of Experimental Data

To decouple the model structure from the corresponding measurement data is good practice in model-based data evaluation. In FluxML the network formulation and the data descriptors are located in different sub-branches of the document tree (cf. Figure 2). This way, model specification and data can be combined in one single document or, alternatively, in two separate files. Principally all measured quantities have to be supplied together with a (strictly positive) measurement error. This measurement uncertainty may refer to data precision or accuracy and cover solely technical or biological uncertainty. Although standard deviations are often based on experiences, such kinds of assumptions are better explicitly documented. In FluxML the description of the measurements and their errors is located within the *data* -branch:

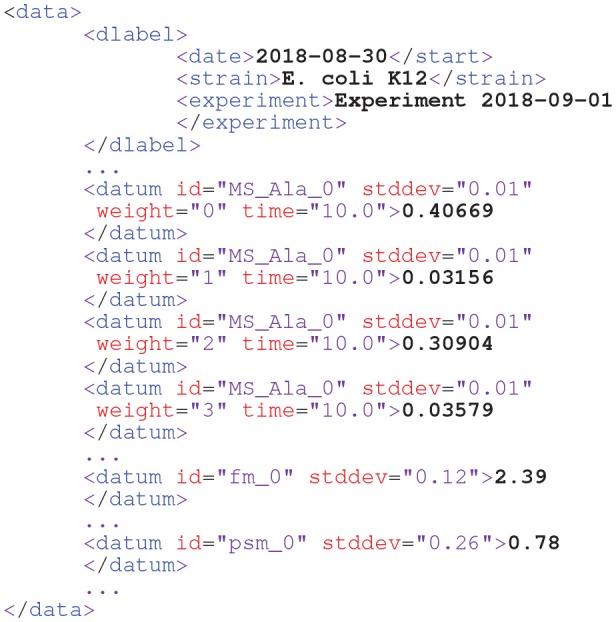



Here, the identifier *MS_Ala_0* refers to the time-resolved MIDs of *ALA*, distinguished by the number of labeled ions contained (*weight*). Each *datum* element specifies exactly one measurement value along with its associated standard deviation (*stddev*) and sampling time point. The measurements for the uptake rate *Glc_upt* (*fm_0*) and the pool size of *ALA* (*psm_0*) are specified similarly.

### Simulation

Whether fluxes and pool sizes are specified as free parameters or being constraint to fixed values impacts flux estimation and the statistical assessment of the final flux map (Heise et al., 2015; Theorell et al., 2017). Thus, the parametrization of the model should also be part of a model. To this end, in FluxML the *variables* element within the simulation branch collects the models' variables (free fluxes and, in case of INST, pool sizes) and their values as well as the minimum information to connect the model description with the simulation framework of choice:

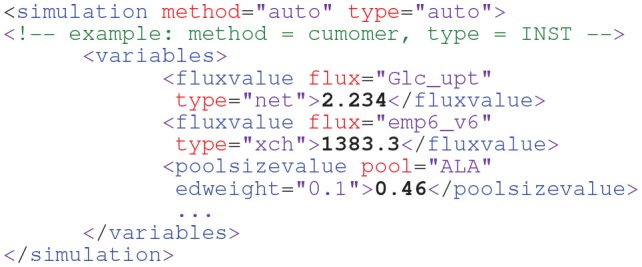



Being designed as a simulator-independent language, details about specific simulation scenarios and settings (solver parametrization, integration times etc.) are intentionally not part of FluxML. For this purpose, scientific workflow, and provenance data description languages have been developed such as CWL [www.commonwl.org/].

### Special Settings for ILE Design

OED of ILEs aims at customizing the experimental settings in a way that the ILE's information gain is maximized. As such, optimal ILE design has become an integral part of ^13^C MFA workflows. Many contemporary software systems provide decision metrics for selecting “informative” tracer mixtures (Möllney et al., 1999; Weitzel et al., 2013; Millard et al., 2014; Shupletsov et al., 2014; Young, 2014). In optimal ILE design, the information gain of an ILE is tested *in silico* by assuming hypothetical experimental-analytical settings. In this context it is important to recognize that OED strategies require not only the measurement model of the envisioned (but physically not yet available) data sets, but also an estimation of their associated standard deviations. Literature mining indicates that errors of labeling enrichments can be heteroscedastic, rather than obeying constant absolute or relative variances (Nöh et al., 2018). For a universal modeling language this implies the need to formulate arbitrary functional dependencies between the “envisioned” measurements and their errors to overcome a lack of real data. How this is solved in FluxML, is exemplified for a tandem-MS measurement group of *ALA*:

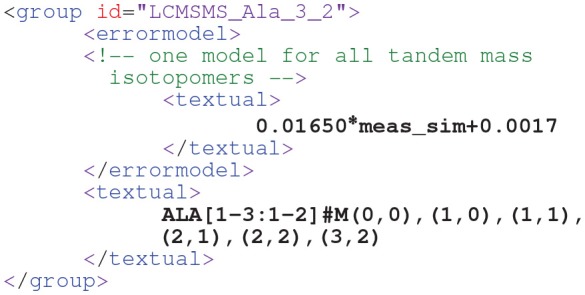



Within the *errormodel* construct, functional expressions derived from analytical expert knowledge relate the simulated measurement values (*meas_sim*), to their associated errors. In an analogous manner, error models for extracellular rate and pool size measurements can be formulated.

Besides a full experimental design, scenarios can be envisioned in which some parameters is given a higher importance than others. The importance of a parameter can be specified by the *edweight* attribute in the *variables* section (cf. listing in Section Simulation, where the pool size of *ALA* is given minor importance compared to the two flux values). In this way, partial experimental designs can be realized (Möllney et al., 1999).

### Housekeeping: Enriching FluxML With Annotations

Developing a model requires documentation which puts the model into the context of the analysis scenario it is built for. FluxML has various dedicated fields to deposit such kind of information. For instance, the top-level *info* element contains the necessary information to achieve MIRIAM-compliance:

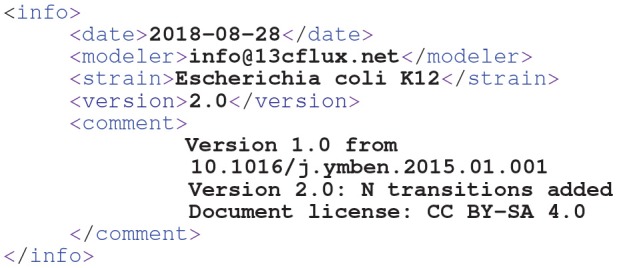



Owing to the configuration concept, also the *data*-branch contains dedicated elements to carry information about the experiment, analytics and data, such as units. Furthermore, annotation elements can be added to any FluxML element, in which XML-compliant content can be stored. For instance, pathway information is helpful to structure comprehensive models or, associating a reactant with its InChI code enables metabolite identification and database matching.

## FluxML Collection and Supporting Tools

Although human-readable, FluxML documents are not made for direct editing by modelers. Additional software tools are necessary to verify, read, write, and edit the information contained in a document, to display its contents in a digestible form, and to check the documents' syntax and semantics. These tools fall into three categories:

1. The FluxML language definition.
2. A FluxML parser to analyze model files according to the rules laid down in the language definition and to check their syntactic and semantic validity, as well as for completeness.
3. Converters and utility tools providing facilities for convenient access of FluxML files.

### FluxML Schema

A language is commonly defined by a formal syntax description (the *grammar*). Accordingly, for each released FluxML Level, the formal syntax is defined in W3C XML Schemas [http://www.13cflux.net/fluxml]. Each XML Schema Definition (XSD) describes the structure of a FluxML document and defines strict syntax rules for the elements and attributes contained. This grammar definition constitutes the essential basis for checking the well-formedness of model files and, therefore, any further FluxML processing procedure. The checking procedure itself is the task of the FluxML parser.

### The FluxML Parser *fmllint*

The parser ***fmllint*** is an error-detection oriented software tool that analyzes the syntactical and semantical validity of FluxML model files according to the rules defined in the associated FluxML Schema. The parser loads a specified FluxML document, traverses through the tree structure and turns the textual representation into a set of objects, the in-memory Document Object Model (DOM) tree [www.w3.org/DOM]. To this end, ***fmllint*** uses the capabilities of the DOM XML parser Xerces-C [www.xerces.apache.org] to perform strict validation according to the XSD file. To facilitate precise semantic model validation, in addition to the grammar, an extensive set of semantic rules is implemented in ***fmllint***. Thus, with parsing, existing document structure inconsistencies and context-sensitive issues are detected and expressive error messages and warnings are reported, mostly along with specific correction suggestions.

**Figure 7:**
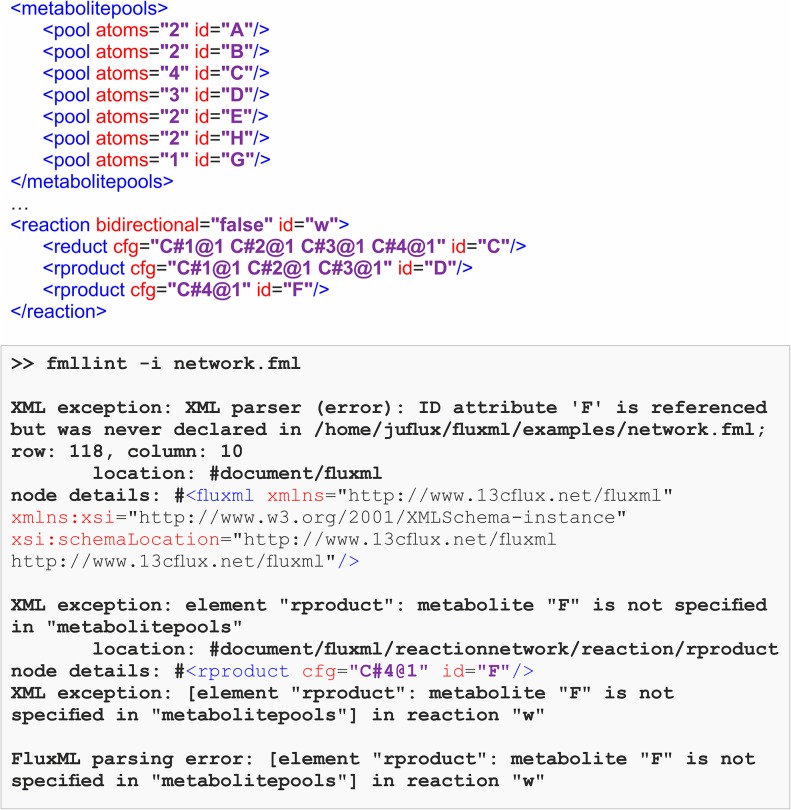
Error backtracking provided by the FluxML parser ***fmllint***. In the FluxML model network.fml, the metabolite pool *F*, which participates in the reaction *w* is not declared in the metabolitepools section. ***fmllint*** provides an expressive error log, pointing to the origin of the error.

Figure 7 gives an example where the metabolite pool *F*, which participates in the reaction *w*, has been forgotten to be specified. Here, during the parsing process, ***fmllint*** detects the missing metabolite *F* and reports an error. The error message provides precise information such as the error location (row and column number) which helps to quickly fix the issue. Several examples, typical for erroneous ^13^C MFA models, are given in the Supplementary S1 Section 3. Some of the most important validity checks of ***fmllint***, specific to ^13^C MFA models are:

- Validation of reaction network, atom transitions and the labeling sources:
  ◦ Missing labeling sources or effluxes
  ◦ Dead-end and disconnected metabolites
  ◦ Traps and isles in the metabolic and atom transition networks
  ◦ Missing metabolite/reaction declarations or duplicates
  ◦ Invalid and elementally imbalanced atom transitions
  ◦ Infeasible/inconsistent input (mixture) specification or purities
- Validation of stoichiometric balances and constraints:
  ◦ Too few/many equality constraints leading to under- or overdetermined stoichiometry
  ◦ Duplicate or linearly dependent equality constraints
  ◦ Infeasible inequality constraints
  ◦ Too few/many free parameters
  ◦ Infeasible parameter values violating the set of constraints
- Validation of the measurements:
  ◦ Missing measurement declarations or duplicates
  ◦ Invalid measurement specification or duplicates
  ◦ Missing and infeasible values for labeling fractions, pool sizes or measurement times
- Validation of the FluxML structure:
  ◦ Missing or invalid XML namespace
  ◦ Well-formedness of textual- or MathML notations
  ◦ Missing or invalid element nodes
  ◦ Invalid attributes or attribute combinations

Currently, in total more than 500 different errors are detected. This number emphasizes the complexity of model specification and the critical importance for having a concise and clean language standard definition. It also demonstrates the complexity and power of the ***fmllint*** parser which is written in ANSI/ISO C/C++ and consists at the time of writing of more than 70 k LOCs (lines of code). The implementation solely depends on standard ANSI C libraries only and is, thus, highly portable.

### Converters and Utilities

#### Tools for Model Reuse

To enable the effective reuse of models was the main driver for developing the FluxML language. To support this goal, the following language translators are supplied:

1. ▪ ***ftbl2fml***: The FTBL-to-FluxML converter conveniently transfers the tabular-separated FTBL format to FluxML Level 1. The converter is implemented in Python and uses the C++ program *expr2mml* to analyze the equality- and inequality-constraints. By employing the ***ftbl2fml*** converter, only minimal steps are needed to transform models built for the ^13^C MFA tools such as influx_s and OpenFLUX into FluxML.
2. ▪ ***fmlupdate***: Modeling languages evolve over time. Therefore, it is important to support modelers with handling changes in language constructs. The FluxML update tool ***fmlupdate*** transforms FluxML documents to new versions. This makes model reuse convenient for end-users when updates of simulation tools inquire newer FluxML versions.
3. ▪ ***sbml2fml, fml2sbml***: Due to their shared language subset, part of the metabolic network and flux constraints can be translated between FluxML and SBML. For easy translation of such network structures, the Python-based converters ***sbml2fml*** and ***fml2sbml*** have been developed.

#### Auxiliary Tools for Everyday Operations

From a users' perspective graphical tools for model building and configuration are preferable. To this end, the comprehensive Fluxomix modeling suite has been developed as plugin-suite for the visualization software Omix (Nöh et al., 2015). However, for integrating model configuration procedures into computational evaluation workflows, programmatic model access is much more convenient than visual modeling. To support commonly performed steps, tools that have been initially released with the 13CFLUX2 software suite, are lifted as standalone Python tools:

▪ **fmlstats**: Summarizes the most important information about the model structure
▪ **setinputs:** Tool for manipulation of the mixture composition of input pools
▪ **setparameters:** Tool to transfer fluxes and/or pool sizes from CSV into FluxML files
▪ **setmeasurements:** Tool for transferring labeling and flux measurements from CSV documents into FluxML files

#### A Software Library for FluxML Tool Developers

The software library ***libFluxML*** is a library for reading, writing and altering FluxML documents. The library provides a rich application programming interface (API) enabling full access to the FluxML language content and a range of functions that facilitate the creation, validation, and manipulation of FluxML documents. ***libFluxML*** offers helper functions for processing and manipulating mathematical formulas in both, human-readable textual notation and machine-readable Content-MathML format, as well as the ability to interconvert mathematical expressions between these forms. Many higher-level convenience features are included, such as for obtaining the number of reactions or constructing the stoichiometric matrix of the reaction network. The library is written in standard ANSI/ISO C/C++ and uses the FluxML parser ***fmllint*** for parsing and validity checking.

### Availability

The FluxML collection consisting of the formal schema definitions, the ***fmllint*** parser, versatile tools and the core library ***libFluxML*** represents an all-inclusive suite to validate and manipulate FluxML documents. Schema files are located at http://www.13cflux.net/fluxml. The source codes of the FluxML parser ***fmllint***, the ***libFluxML*** library, and the auxiliary tools are available at the github repository https://github.com/modsim/FluxML/ with full built instructions, comprehensive documentation and usage examples. In addition, precompiled binary distributions for Linux and Mac OS X are provided. The FluxML collection is licensed under the open-source Creative Commons Attribution-ShareAlike (CC BY-SA 4.0)^5^ and MIT^6^ licenses. In addition, for model checking without installation, a web-based FluxML validator is available at http://www.13cflux.net/fluxml/validator/. Altogether, this collection provides a set of tools for interfacing and validating FluxML documents and, as such, provides a solid tool base for future developments of the modeling language FluxML.

## Harnessing the Benefits of FluxML

Finally, we give two examples for the utility and usability of the FluxML language. We first illustrate how using one single model, formulated in FluxML, can be used with different ^13^C MFA tools to facilitate the comparison of results. Secondly, we demonstrate how parallel ILEs are efficiently modeled starting from a single ILE setup.

### FluxML for Simulator Comparisons

From a users' perspective, the lack of abilities to compare and validate numerical results generated by different ^13^C MFA tools is unsatisfactory. Clearly, a precise and unambiguous representation of a model provides the basis for any of these tasks. Extracting the encoding of a model formulated for one piece of software and transferring it to another format is a step prone to errors that should be subjected to converters. Here, we exemplify a simulator comparison, taking the deterministic forward simulation step with 13CFLUX2 (v2.0) and Sysmetab (v5.1, Mottelet et al., 2017) as representative test case. The comparison is done with a central metabolism model of *E. coli* contained in the Sysmetab distribution, precisely, a isotopically stationary and non-stationary variant mimicking ILEs with a 3:7 [U-^13^C]:[1-^13^C]-glucose mixture. The ***fmlstats*** tool reports that the network consists of 51 metabolites and 86 reactions. In total 9 MS measurement groups and one extracellular flux measurement are contained.

**Figure 8:**
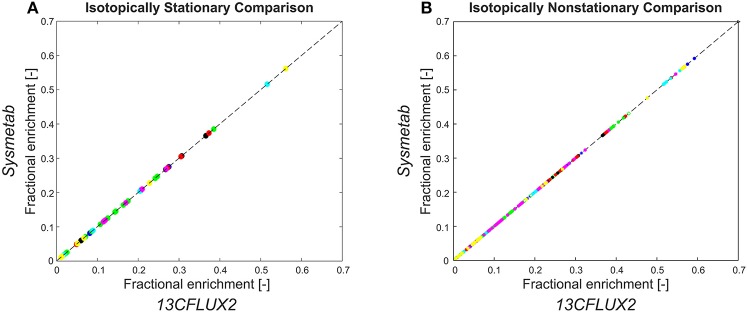
Parity plots showing simulation results generated by 13CFLUX2 and Sysmetab. Classical, isotopically stationary approach **(A)** and INST ^13^C MFA approach **(B)**. One color is used per measurement group. For comparability, both simulators were configured to use the same labeling framework, i.e., the reduced cumomers (Weitzel et al., 2007). Both simulators use sparse LU factorization for solving the cascaded cumomer systems. The differential equation systems were solved using Sundial's CVODE solver [https://computation.llnl.gov/projects/sundials/cvode/] (13CFLUX2) and a 4th order singly diagonally implicit Runge-Kutta method (Sysmetab). Both solvers used adaptive stepsize control with error tolerance (10^−6^). Simulation results and scripts for generating the parity plots are available in the Supplementary Data Sheet 2.

In the classic isotopically stationary case, the corresponding Sysmetab FluxML was conform with the FluxML Level 1 definition. Both simulators were invoked and simulated labeling patterns extracted from the tools' output. The comparison of the simulated fractional enrichments shows perfect agreement (cf. Figure 8A). For the isotopically non-stationary case, it turned out that the model shipped with Sysmetab lacks pool sizes and is, thus, syntactically invalid with respect to the Level 2 specification for INST ^13^C MFA. Since Sysmetab internally allocates positive random values to the pool sizes in the simulation step, these model parameters had to be extracted from the simulation output. After updating the INST FluxML model to conform with the Level 2 Schema definition using the ***fmlupdate*** tool, the pool sizes were incorporated into the file utilizing the ***setparameters*** command. Lastly, the 13CFLUX2 simulator was called to execute the simulation step. Again, the simulated MIDs for the nine measurement groups produced by the two simulators were extracted from the output files and plotted in a parity plot, showing excellent agreement (cf. Figure 8B). This example shows the importance of syntactic model validation in view of reporting standards. Besides a clear and complete language definition, appropriate converters, and auxiliary tools are needed to tame the zoo of available model files, often developed specific to ^13^C MFA software systems.

### Parallel Labeling Experiments

Experimental design has been an essential part of the general flux analysis workflow since the beginnings of ^13^C MFA. As such, numerous studies investigated how specific experimental configurations, predominantly the input tracers, and substrate mixtures, but also the number, type and quality of measurements influence the statistical quality of the flux estimates. To increase the information gain about fluxes, it has been suggested to use multiple experiments operated under exactly the same physiological conditions, each with a different tracer (Schwender et al., 2006; Antoniewicz, 2015). The evaluation of such, so called, parallel ILEs (pILE) requires the modeler to merge all data sets in one measurement specification. By expressing pILEs in the FluxML language, their simulation can be readily achieved by employing standard ^13^C MFA tools. In particular, we show that the evaluation of pILEs becomes a special case of the traditional single ILE-based ^13^C MFA.

The general principle is in fact simple: When *N* different experiments are performed (in practice usually up to tens), one option is that the modeler supplies the original network formulation in *N* multiple copies. In each of the copies all metabolites and reactions are multiplied (practically by appending their identifiers with an additional suffix relating them to the experiment to which their associated measurement sets). In addition, for each flux (and pool size, in the case of INST) an additional constraint must be specified in the FluxML document which assures that the values of the model parameters are the same for all network copies^7^. Clearly, to perform these operations manually is laborious and means to pay painstaking attention to details.

By using the available FluxML capabilities, automation of this operation is straightforward. To this end, the program ***multiply_fml*** was implemented with only 400 single LOCs (SLOC) of Python code. ***multiply_fml*** expects an FluxML file with *N* configurations each equipped with the input specification and a corresponding measurement set for one ILE. The duplication process is showcased with 14 different ILEs with the setting reported by Crown et al. (2015). First, the different input mixtures are specified one-at-a-time in 14 different configurations of the reference network by invoking the ***setinputs*** function:


setinputs -i crown.fml -c config_01 -C
input_01.csv -o crown.fml


etc. Secondly, the measurement data sets are sequentially incorporated by using the ***setmeasurements*** tool:


setmeasurements -i crown.fml -c config_01
-C data_01.csv -o crown.fml

Finally, the network duplication step is performed using the ***multiply_fml*** program:


multiply_fml -i
crownl.fml -o crown_multiplied.fml


The resulting model file (crown_multiplied.fml) consists of one network description and a single configuration comprising all 14 ILE data sets (all FluxML and CSV files used in this showcase are available in the Supplementary Data Sheet 3). With this model at hand, all ^13^C MFA tools can be invoked. For example, optimal tracer design for a series of pILEs is possible, rephrased as the choice of the best substrates per experiment. This makes application of experimental design tools of ^13^C MFA software straightforward.

## Conclusion

^13^C MFA is the primary experimental technique for measuring intracellular fluxes at metabolic pseudo-steady state conditions. After two decades of active research there is consensus about the minimal information set needed to specify a computational ^13^C model and its associated data. However, this consensus has not yet found its way into a model format that contains the complete information set of an ILE configuration in a well-structured manner. Most importantly, implicit assumptions made in the modeling process are rarely included in publications because they are considered to be common sense or of purely technical nature. This makes it essentially impossible to reproduce many published flux analysis results.

On the one hand, the complexity and depth of ILE specifications should not hinder experimentalists to deliver complete ^13^C MFA models. In this context, it is of great advantage that tailored model templates can be configured (often only once for an organism or strain) whereas experiment specific data is fed into these templates using preconfigured scripts. For power users, on the other hand, computational model components should be programmatically accessible, so that they are embeddable in computational pipelines.

Following these two guiding principles, here we describe the Flux Markup Language FluxML along with its design. The major aim of FluxML is to offer a sound universal, open source, simulator-independent, and future-proof platform that conserves all the necessary and optional information for model description, reuse, exchange and comparison. Specifically, FluxML enables practitioners to describe valid isotopically stationary and non-stationary models, while the format is fully universal in term of network, atom mapping, measurement (error) and constraint formulation, including the use of homo- and hetero-isotopic tracers. With the language, the FluxML collection is supplied which contains the powerful FluxML parser ***fmllint*** for model (in)validation and several auxiliary tools for easy handling, but also to allow for a maximum of flexibility. We believe that FluxML improves scientific productivity, efficiency as well as transparency and contributes to the reproducibility of computational modeling efforts in the field of ^13^C MFA.

## Author Contributions

WW and KN conceived the work. MW developed FluxML Level 1. SA and MB developed Levels 2/3 and performed the computational analyses. WW and KN wrote the manuscript to which SA and MB contributed. All authors approved the content of the manuscript.

### Conflict of Interest Statement

The authors declare that the research was conducted in the absence of any commercial or financial relationships that could be construed as a potential conflict of interest.

## Footnotes

^1^The use of ^13^C labeled tracers is the mainstream scenario for ^13^C MFA. The utilization of other stable isotope labeling strategies such as ^15^N or simultaneous hetero-isotopic tracer combinations such as ^13^C-^15^N is conceptually equivalent. Since it is the established notion in the field, the term “^13^C MFA” is used throughout, although we extend its meaning to all of these alternative labeling strategies.

^2^“In particular, it is critical that data essential to a model definition … is not stored in annotations” [http://sbml.org/special/specifications/sbml-level-3/version-2/core/release-1-rc1/sbml-level-3-version-2-core.pdf].

^3^Notice that a simple permutation transforms the atom positions in the biochemical lettering and the InChI-based canonical enumeration into one another.

^4^Note that in classical ^13^C MFA the fluxes can be scaled arbitrarily, as long as they share the same units.

^5^https://creativecommons.org/licenses/by-sa/4.0/legalcode

^6^https://opensource.org/licenses/mit-license.php

^7^Another option is to double the metabolites and add the pool copies to the reactions, along with the formulation of pool size constraints in case of INST.

## Abbreviations

FluxML: Flux Markup Language
FTBL: Flux TaBuLar format
ILE: isotope labeling experiment
INST: isotopically non-stationary
MFA: metabolic flux analysis
MID: mass isotopomer distribution
MS: mass spectrometry
NMR: nuclear magnetic resonance
OED: optimal experimental design
(S)LOC: (single) lines of code.
