# Corrigendum: The Design of FluxML: A Universal Modeling Language for ^13^C Metabolic Flux Analysis

**DOI:** 10.3389/fmicb.2019.01734

**Published:** 2019-07-30

**Authors:** Martin Beyß, Salah Azzouzi, Michael Weitzel, Wolfgang Wiechert, Katharina Nöh

**Affiliations:** ^1^Institute of Bio- and Geosciences, IBG-1: Biotechnology, Forschungszentrum Jülich GmbH, Jülich, Germany; ^2^Computational Systems Biotechnology (AVT.CSB), RWTH Aachen University, Aachen, Germany

**Keywords:** ^13^C metabolic flux analysis, FluxML, machine-readable format, model specification language, computational modeling, reproducible science, data models, model exchange

In the original article, Sysmetab was cited by “Mottelet et al. 2017” in Supplementary S1 Table 1.1 and not in the main text of the article. The citation has now been inserted in the section Harnessing the Benefits of FLUXML, sub-section FluxML for Simulator Comparisons, paragraph one:

“From a users' perspective, the lack of abilities to compare and validate numerical results generated by different ^13^C MFA tools is unsatisfactory. Clearly, a precise and unambiguous representation of a model provides the basis for any of these tasks. Extracting the encoding of a model formulated for one piece of software and transferring it to another format is a step prone to errors that should be subjected to converters. Here, we exemplify a simulator comparison, taking the deterministic forward simulation step with 13CFLUX2 (v2.0) and Sysmetab (v5.1, Mottelet et al., 2017) as representative test case. The comparison is done with a central metabolism model of *E. coli* contained in the Sysmetab distribution, precisely, a isotopically stationary and non-stationary variant mimicking ILEs with a 3:7 [U-^13^C]:[1-^13^C]-glucose mixture. The ***fmlstats*** tool reports that the network consists of 51 metabolites and 86 reactions. In total 9 MS measurement groups and one extracellular flux measurement are contained.”

The authors apologize for this error and state that this does not change the scientific conclusions of the article in any way. The original article has been updated.
