# Supplementary material for: The Design of FluxML: A Universal Modeling Language for 13C Metabolic Flux Analysis
Source: Front Microbiol. 2019 May 24;10:1022. doi: 10.3389/fmicb.2019.01022 (PMC6543931; doi:10.3389/fmicb.2019.01022)
Supplement: Supplementary file 1 [file Data_Sheet_1.PDF]

Supplementary Material

The Design of FluxML: A Universal Specification Language for ^{13}C Metabolic Flux Analysis

Martin Beyß[♦,1], Salah Azzouzi[♦,1], Michael Weitzel[♦,1], Wolfgang Wiechert[♣,1,2], Katharina Nöh[♣,1,*]

♦,♣ Equal contribution

[1]Institute of Bio- and Geosciences, IBG-1: Biotechnology, Forschungszentrum Jülich GmbH, Jülich, Germany

[2]Computational Systems Biotechnology (AVT.CSB), RWTH Aachen University, Aachen, Germany

*** Correspondence:** k.noeh@fz-juelich.de

Contents

1 Selected publicly available software systems for ^{13}C MFA 2

2 UML diagram of FluxML 5

3 Typical FluxML error messages and warnings 6

4 Miscellanea 10

4.1 A use case for Content-MathML 10

4.2 Scrambling reactions: conventional vs. variant formulation 11

4.3 Hetero-isotopic input substrates (Level 3) 12

4.4 Exchange of substances across the cell boundary 13

4.5 Hetero-isotopic mass spectrometric data specification (Level 3) 14

1 Selected publicly available software systems for ^{13}C MFA

Supplementary Table 1.1. Collection of software tools for ^{13}C MFA and their modeling format-related features (sorted by publication date).

Software	Type	Programing language	Model specification interface, Document type/format	Comments	Citation
13CFLUX<br>Siegen University, Siegen, Germany<br>2001	Classical	C++	Text-based,<br>FTBL	Meas. type: universal<br>Atom mappings: letter<br>State var.s: Cumomer	doi:<br>10.1006/mben.2001.0188
tcaSIM, tcaCALC<br>Southwestern University<br>Georgetown, Texas<br>2004	Classical	DOS	n.a. (fixed model)	Meas. types: NMR, MS<br>Atom mappings: n.a.<br>State var.s: isotopomers	doi:<br>10.1016/j.ymben.2003.10.007
FIATFLUX (SumoFlux)<br>ETH Zurich<br>Zurich, Switzerland<br>2005 (2016)	Flux ratios	Matlab	GUI,<br>text-based, netCDF (data),<br>MAT-file	Meas. type: MS<br>Atom mappings: n.a.<br>(preconfigured flux ratios for [1-^{13}C]- and [U-^{13}C])	doi:<br>10.1371/journal.pcbi.1005109
METRAN<br>MIT, Cambridge, Massachusetts,<br>University of Delaware<br>Newark, Delaware<br>2005	Classical	Matlab	GUI<br>text-based, MAT-file	Meas. type: NMR, MS<br>Atom mappings: letter<br>State var.s: EMU	doi:<br>10.1074/jbc.M706494200
OpenFlux<br>University of Queensland<br>Brisbane, Australia<br>2009	Classical	Matlab	Text-based,<br>FTBL derivate	Meas. type: MS<br>Atom mappings: letter<br>State var.s: EMU	doi:<br>10.1186/1475-2859-8-25

C13<br>Chalmers University of Technology, Göteborg, Sweden<br>2010	Classical	Matlab	Text-based,<br>CSV	Meas. type: MS<br>Atom mappings: letter<br>State var.s: Isotopomer	doi:<br>10.1093/nar/gkq404
influx_s / influx_si<br>Université de Toulouse<br>Toulouse, France<br>2012, 2014	Classical,<br>INST	R, Python	Text-based,<br>FTBL	Meas. type: universal<br>Atom mappings: letter<br>State var.s: Cumomer, EMU	doi:<br>10.1093/bioinformatics/btr716
13CFLUX2<br>Forschungszentrum Jülich<br>Jülich, Germany<br>2012	Classical,<br>INST	C++	GUI via Omix,<br>Text-based,<br>FluxML	Meas. type: universal<br>Atom mappings: INCHI, letter<br>State var.s: Cumomer, EMU	doi:<br>10.1093/bioinformatics/bts646
INCA<br>Vanderbilt University<br>Nashville, Tennessee<br>2013	Classical,<br>INST	Matlab	GUI,<br>MAT-file	Meas. type: MS<br>Atom mappings: letter<br>State var.s: EMU	doi:<br>10.1093/bioinformatics/btu015
OpenFlux2<br>Ajinomoto-Genetika Research Institute,<br>Mosow, Russia<br>2014	Classical	Matlab	Text-based,<br>FTBL derivate	Meas. type: MS<br>Atom mappings: letter<br>State var.s: EMU	doi:<br>10.1186/s12934-014-0152-x
OpenMebius<br>Osaka University<br>Osaka, Japan<br>2014	INST	Matlab	Text-based,<br>CVS	Meas. type: MS<br>Atom mappings: letter<br>State var.s: EMU	doi:<br>10.1155/2014/627014
WUFlux<br>Washington University<br>St. Louis, Missouri<br>2016	Classical	Matlab	GUI, text-based,<br>MAT-file, CSV	Meas. type: MS<br>Atom mappings: letter<br>State var.s: EMU	doi:<br>10.1186/s12859-016-1314-0

Sysmetab<br>Sorbonne University<br>Paris, France<br>2017	Classical,<br>INST	Scilab	Text-based,<br>FluxML	Meas. type: universal<br>Atom mappings: letter<br>State var.s: Cumomer	doi:<br>10.1109/TCBB.2016.2544299
jQMM<br>Lawrence Berkeley<br>National Laboratory<br>Emeryville,<br>California<br>2017	Classical	Python	Text-based,<br>SBML	Meas. type: MS<br>Atom mappings: letter<br>State var.s: EMU	doi:<br>10.1186/s12859-017-1615-y
FluxPyt<br>International Centre<br>for Genetic<br>Engineering and<br>Biotechnology<br>New Delhi, India<br>2018	Classical	Python	Text-based,<br>FTBL derivate	Meas. type: MS<br>Atom mappings: letter<br>State var.s: EMU	doi:<br>10.7717/peerj.4716

2 UML diagram of FluxML

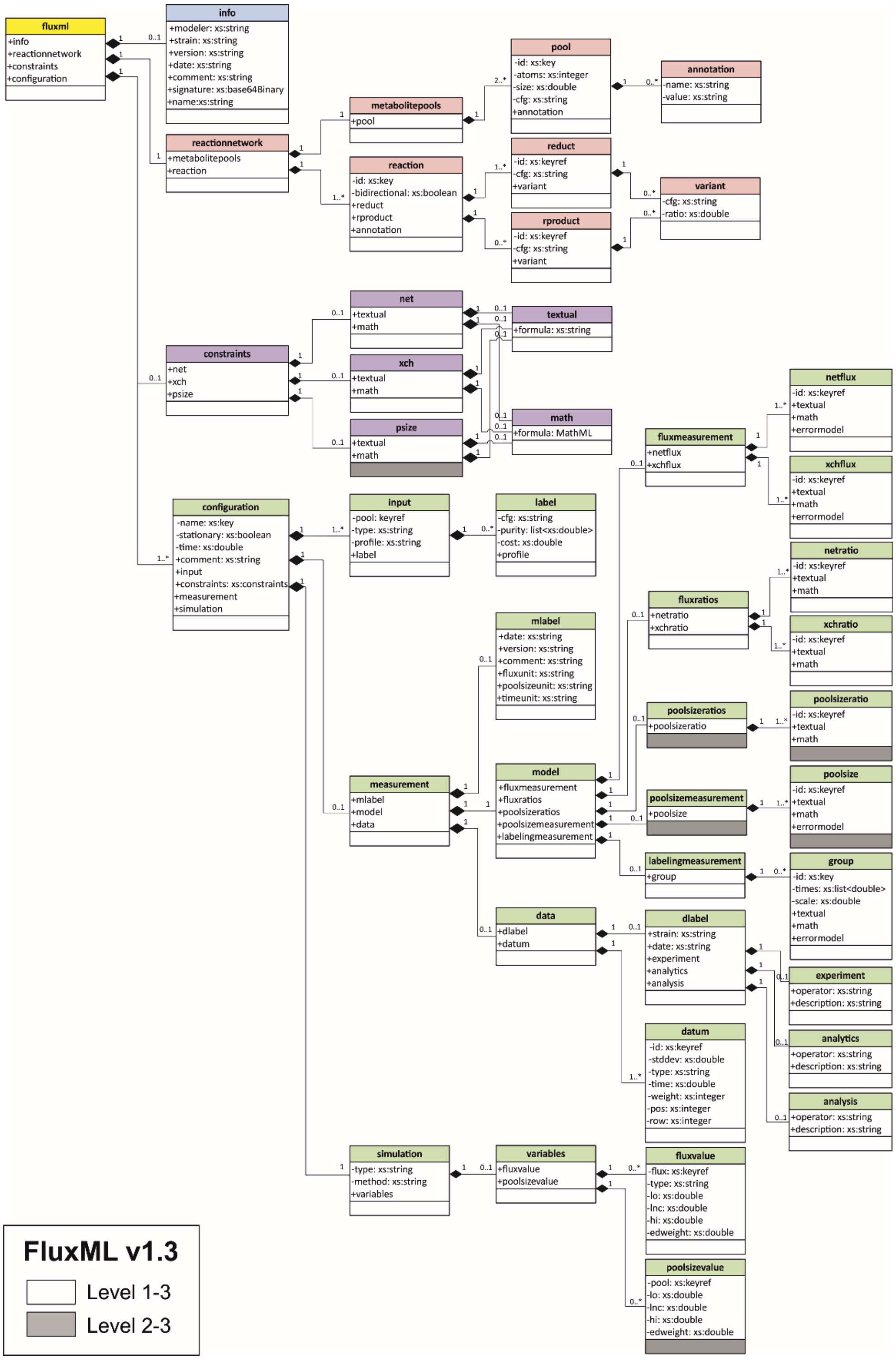

Supplementary Figure 2.1. Class diagram of FluxML data structures (FluxML v1.3) in UML (ISO/IEC 19505). The full XML Schema Definition (XSD) is found at www.13cflux.net/fluxml.

3 Typical FluxML error messages and warnings

Example 1: Erroneous atom mappings and the corresponding error message reported:

```
<reaction bidirectional="false" id="w">
   <reduct cfg="C#1@1 C#2@1 C#3@1 C#4@1" id="C"/>
   <rproduct cfg="C#2@1 C#3@1 C#5@1" id="D"/>
   <rproduct cfg="C#1@1" id="F"/>
</reaction>
```

```
>> fmllint -i Spirallus.fml

illegal permutation -- C#4@1 unmatched in cfg of reaction w
XML exception: reaction "w": illegal permutation in atom transitions

      location: #document/fluxml/reactionnetwork/reaction
node details: #<reaction bidirectional="false" id="w"/>

FluxML parsing error: reaction "w": illegal permutation in atom transitions
```

Example 2: Number of atoms not matching:

```
<pool atoms="2" id="F"/> [...]
<reaction bidirectional="false" id="w">
   <reduct cfg="C#1@1 C#2@1 C#3@1 C#4@1" id="C"/>
   <rproduct cfg="C#2@1 C#3@1 C#4@1" id="D"/>
   <rproduct cfg="C#1@1" id="F"/>
</reaction>
```

```
>> fmllint -i Spirallus.fml

element "rproduct" (id="F"): "cfg" attribute shows 1 labeling positions whereas pool definition specifies 2 labeling postions

          location: #document/fluxml/reactionnetwork/reaction/rproduct
    node details: #<rproduct cfg="C#4@1" id="F"/>
XML exception: [element "rproduct" (id="F"): "cfg" attribute shows 1 labeling positions whereas pool definition specifies 2 labeling postions] in reaction "w"

FluxML parsing error: [element "rproduct" (id="F"): "cfg" attribute shows 1 labeling positions whereas pool definition specifies 2 labeling postions] in reaction "w"
```

Example 3: A non-existing reaction is referred to in the constraints:

```
<reaction bidirectional="false" id="u">…</reaction>
<reaction bidirectional="false" id="v">…</reaction>
<reaction bidirectional="false" id="w">…</reaction>
<reaction bidirectional="false" id="p">…</reaction>
<reaction bidirectional="true" id="q">…</reaction>
<reaction bidirectional="false" id="r">…</reaction>
…
<constraints>
  <net>
    <textual> u &lt;= 1; a= 1; <textual>
  </net>
</constraints>
```

```
>> fmllint -i Spirallus.fml

XML exception: element XML exception: invalid reaction name "a" in constraint "anonymous" (a=1)

    location: #document/fluxml/constraints/net/textual/#text
node details: #text{u <=1; a=1;}

FluxML parsing error: invalid reaction name "a" in constraint "anonymous" (a=1)
```

Example 4: Forgotten tags <textual> </textual> in the net constraints section:

```
<constraints>
  <net>
    u &lt;= 1;
  </net>
</constraints>
```

```
>> fmllint -i Spirallus.fml

XML exception: element XML XML exception: XML parser (error): no character data is allowed by content model in /home/fluxml/examples/Spirallus.fml;
row: 53, column: 10
      location: #document/fluxml/constraints/net
  node details: #<net/>

XML exception: XML parser (error): no character data is allowed by content model in /home/fluxml/examples/Spirallus.fml; row: 54, column : 1
      location: #document/fluxml/constraints/net
  node details: #<net/>

XML exception: XML parser (error): empty content is not valid for content model '(textual|math)' in /home/fluxml/examples/Spirallus.fml; row: 54, column: 7
     location: #document/fluxml/constraints/net
  node details: #<net/>
XML exception: net constraints in textual- or MathML notation expected
location: node details: (null)

FluxML parsing error: net constraints in textual- or MathML notation expected
```

Example 5: Incomplete XML tree; missing tags in “xch constraints” section:

```
<fluxml>
  <reactionnetwork> …. </reactionnetwork>
  <constraints>
    <xch> q &lt;= 0.7; </xch>
  <configuration > ... </configuration>
```

```
>> fmllint -i Spirallus.fml

XML exception: XML parser (fatal error): expected end of tag 'xch' in /home/
fluxml/examples/Spirallus.fml; row: 58, column: 3
location: #document/fluxml/constraints/xch/#text
node details: #text{ }

or

XML exception: element XML xmllint Spirallus.fml
Spirallus.fml:58: parser error : Opening and ending tag mismatch: xch line 55 and
constraints
</constraints>

Spirallus.fml:118: parser error : Opening and ending tag mismatch:
constraints line 51 and fluxml
</fluxml>

Spirallus.fml:119: parser error : Premature end of data in tag fluxml line 2
```

Example 6: “xch constraints” section not properly closed:

```
<constraints>
  <net>
    <textual> u &lt;=1;</textual>
  </net>
  <xch>
    <textual>q &lt;=1</textual>
</constraints>
```

```
>> fmllint -i Spirallus.fml

XML exception: XML exception: XML parser (fatal error): expected end of tag 'fluxml' in
/home/fluxml/examples/Spirallus.fml; row: 115, column: 3
    location: #document/fluxml/#text
node details: #text{ }

XML exception: element node (configuration) expected.
location: #document/fluxml/comment node details: #<comment/>

FluxML parsing error: element node (configuration) expected.
```

Example 7: Isolated sub-network detected (typically isolated nodes or sub-networks are unintentionally introduced during the modeling process and not recognized by the modeler):

```
<reaction> … </reaction>

<!-- isolated-subnetwork -->
<reaction bidirectional="false" id="h">
    <reduct cfg="C#1@1 " id="H"/>
    <rproduct cfg="C#1@1 " id="G"/>
</reaction>
```

```
>> fmllint -i Spirallus.fml

pool "H" has no efflux (set at least one incident exchange flux >0) missing input pool specification (cfg: "default", pool: "G") XML exception: validation of configuration default failed!

FluxML parsing error: validation of configuration default failed!
```

4 Miscellanea

4.1 A use case for Content-MathML

The measurement group of the tandem MS measurement fragment *ALA[1-3:1-2]* with measurement error model

```
meas_sim*(0.000780+(meas_sim^(-0.739716))/(40.001740+meas_sim^(-0.739316)))
```

reads, using Content-MathML, as follows:

```
<group id="LCMSMS_Ala_3_2">
      <errormodel>
            <math xmlns="http://www.w3.org/1998/Math/MathML">
                  <apply>
                        <times/>
                        <ci>meas_sim</ci>
                        <apply>
                              <plus/>
                              <cn type="e-notation">7.8<sep/>-4</cn>
                              <apply>
                                    <divide/>
                                    <apply>
                                          <power/>
                                          <ci>meas_sim</ci>
                                          <apply>
                                                <minus/>
                                                <cn type="real">0.739716</cn>
                                          </apply>
                                    </apply>
                                    <apply>
                                          <plus/>
                                          <cn type="real">40.00174</cn>
                                          <apply>
                                                <power/>
                                                <ci>meas_sim</ci>
                                                <apply>
                                                      <minus/>
                                                      <cn type="real">
                                                                  0.739316</cn>
                                                </apply>
                                          </apply>
                                    </apply>
                              </apply>
                        </apply>
                  </apply>
            </math>
            </errormodel>
      <textual>
            ALA[1-3:1-2]#M(0,0),(1,0),(1,1),(2,1),(2,2),(3,2)
      </textual>
</group>
```

4.2 Scrambling reactions: Conventional vs. variant formulation

The following two listings show the formulation of the symmetric succinate dehydrogenase reaction step in the tricarboxylic acid cycle, first with using the dedicated *<variant/>* modeling elements and second in the traditional way by specification of two reactions, whose fluxes are explicitly equitized.

a) Formulation via `variant` elements:

```
<reaction bidirectional="true" id="TCA7_v26_1 TCA7_v26_2">
      <annotation name="pathway">TCA</annotation>
      <reduct cfg="C#1@1 C#2@1 C#3@1 C#4@1" id="Suc"/>
      <rproductid="Fum">
            <variant cfg=C#1@1 C#2@1 C#3@1 C#4@1" ratio="0.5"/>
            <variant cfg=C#2@1 C#1@1 C#4@1 C#3@1" ratio="0.5"/>
      </rproduct>
      <rproduct cfg="" id="FADH2"/>
</reaction>
```

b) Explicit formulation:

```
<reaction bidirectional="true" id="TCA7_v26_1">
      <annotation name="pathway">TCA</annotation>
      <reduct cfg="C#1@1 C#2@1 C#3@1 C#4@1" id="Suc"/>
      <rproduct cfg="C#1@1 C#2@1 C#3@1 C#4@1" id="Fum"/>
      <rproduct cfg="" id="FADH2"/>
</reaction>

<reaction bidirectional="true" id="TCA7_v26_2">
      <annotation name="pathway">TCA</annotation>
      <reduct cfg="C#1@1 C#2@1 C#3@1 C#4@1" id="Suc"/>
      <rproduct cfg="C#2@1 C#1@1 C#4@1 C#3@1" id="Fum"/>
      <rproduct cfg="" id="FADH2"/>
</reaction>

<constraints>
      <net>
            <textual>
                  TCA7_v26_1=TCA7_v26_2;
            </textual>
      </net>
      <xch>
            <textual>
                  TCA7_v26_1=TCA7_v26_2;
            </textual>
      </xch>
</constraints>
```

4.3 Hetero-isotopic input substrates (Level 3)

The listing shows the formulation of tracers for multi-element ILEs. In this example, the C5-N2 metabolite glutamine (*GLN*) is used as tracer. Specifically, a 2:3 mixture of [$^{13}C_5$,^{14}N]- and [$^{13}C_5$,$^{15}N_2$]-glutamine (*GLN*) is specified, including purities and labeling costs:

```
<input pool="GLN_ext" type="isotopomer">
      <!-- element order C-N, as defined in metabolitepools -->
      <!-- InChI numbering GLN CCCCCNN, as defined in metabolitepools -->
      <!-- costs in EUR/g -->
      <label cfg="1111100" purity="0.98 0.95" cost="6218.0">0.4</label>
      <label cfg="1111111" purity="0.98 0.98" cost="3938.0">0.6</label>
</input>
```

4.4 Exchange of substances across the cell boundary

Substances are not always exclusively taken up or produced. This might be the case for CO_2 or rich media components such as amino acids. As soon as significant exchange between intracellular and extracellular metabolites is present or suspected, this exchange has to be adequately modelled to not bias the interpretation of the observed labeling patterns. The following listing shows how exchange of metabolites is modelled, with CO_2 as example:

```
<!-- exchange of unlabeled CO2 with the environment -->
<!-- specify input pool -->
<input pool="CO2_unlabeled_in" type="isotopomer">
      <label cfg="0">1.0</label>
</input>

...

<!-- specify CO2-exchange reaction with the environment -->
<reaction bidirectional="false" id="CO2_unlabled_upt">
      <reduct cfg="C#1@1" id="CO2_unlabeled_in"/>
      <rproduct cfg="C#1@1" id="CO2_aux"/>
</reaction>
<reaction bidirectional="false" id="CO2_exchange">
      <reduct cfg="C#1@1" id="CO2"/>
      <reduct cfg="C#1@2" id="CO2_aux"/>
      <rproduct cfg="C#1@1" id="CO2_out"/>
      <rproduct cfg="C#1@2" id="CO2"/>
</reaction>
<reaction id="CO2_out">
      <reduct cfg="C#1@1" id="CO2_out"/>
</reaction>
```

4.5 Hetero-isotopic mass spectrometric data specification (Level 3)

The listings exemplify how low and high resolution measurements for the C6-N2 amino acid lysine (*LYS*), generated in a simultaneous ^{13}C-^{15}N ILE, are formulated in FluxML:

```
<!— InChI numbering LYS CCCCCCNN, as defined in metabolitepools -->
<group id="ms_group_LYS_1">
      <!-- MS measurement without C-N resolution -->
      <textual>LYS[1-8]#M0,1,2,3,4,5,6,7,8</textual>
</group>

...

<group id="ms_group_LYS_2">
      <!-- MS measurement with C-N resolution, e.g. Orbitrap MS -->
      <textual>LYS[1-8]#M(0,0),(0,1),(1,0),(0,2),(1,1),
            (2,0),(1,2),(2,1),(3,0),(2,2),(3,1),(4,0),
            (3,2),(4,1),(5,0),(4,2),(5,1),(6,0),(5,2),
            (6,1),(6,2)
      </textual>
</group>
```

Notice the syntactical difference between the specification of hetero-isotopic mass spectrometric measurements, as shown here, and the notation of conventional tandem mass isotopomers as defined in FluxML (Level 1-3), given in Sec. 4.7.2.2 of the main text. In case of the latter, a *pair of fragments* is specified in squared brackets, while in the former case solely the observed metabolite fragment is specified.
